# Application of dual reading domains as novel reagents in chromatin biology reveals a new H3K9me3 and H3K36me2/3 bivalent chromatin state

**DOI:** 10.1186/s13072-017-0153-1

**Published:** 2017-09-25

**Authors:** Rebekka Mauser, Goran Kungulovski, Corinna Keup, Richard Reinhardt, Albert Jeltsch

**Affiliations:** 1 Department of Biochemistry, Institute of Biochemistry and Technical Biochemistry, Stuttgart University, Allmandring 31, 70569 Stuttgart, Germany; 2 Max-Planck-Genomzentrum Köln, Carl-von-Linné-Weg 10, 50829 Cologne, Germany

**Keywords:** Histone code, Multivalent readout, Reading domains, Epigenetics, Chromatin

## Abstract

**Background:**

Histone post-translational modifications (PTMs) play central roles in chromatin-templated processes. Combinations of two or more histone PTMs form unique interfaces for readout and recruitment of chromatin interacting complexes, but the genome-wide mapping of coexisting histone PTMs remains an experimentally difficult task.

**Results:**

We introduce here a novel type of affinity reagents consisting of two fused recombinant histone modification interacting domains (HiMIDs) for direct detection of doubly modified chromatin. To develop the method, we fused the MPP8 chromodomain and DNMT3A PWWP domain which have a binding specificity for H3K9me3 and H3K36me2/3, respectively. We validate the novel reagent biochemically and in ChIP applications and show its specific interaction with H3K9me3–H3K36me2/3 doubly modified chromatin. Modification specificity was confirmed using mutant double-HiMIDs with inactivated methyllysine binding pockets. Using this novel tool, we mapped coexisting H3K9me3–H3K36me2/3 marks in human cells by chromatin interacting domain precipitation (CIDOP). CIDOP-seq data were validated by qPCR, sequential CIDOP/ChIP and by comparison with CIDOP- and ChIP-seq data obtained with single modification readers and antibodies. The genome-wide distribution of H3K9me3–H3K36me2/3 indicates that it represents a novel bivalent chromatin state, which is enriched in weakly transcribed chromatin segments and at ZNF274 and SetDB1 binding sites.

**Conclusions:**

The application of double-HiMIDs allows the single-step study of co-occurrence and distribution of combinatorial chromatin marks. Our discovery of a novel H3K9me3–H3K36me2/3 bivalent chromatin state illustrates the power of this approach, and it will stimulate numerous follow-up studies on its biological functions.

**Electronic supplementary material:**

The online version of this article (doi:10.1186/s13072-017-0153-1) contains supplementary material, which is available to authorized users.

## Background

The fundamental organizational unit of the eukaryotic genome is the nucleosome, which consists of 147 base pairs of DNA wrapped around a histone octamer containing two copies of the histone proteins H3, H4, H2A and H2B. The core histone proteins project out unstructured N-terminal tails, which are subject to more than a hundred post-translational modifications (PTMs) [1, 2]. These histone modifications have instructive roles and directly modulate numerous chromatin-templated processes such as gene expression, DNA replication and repair, as well as normal development and disease [3–5]. The biological effects of histone PTMs are predominantly mediated by a large variety of so-called reading domains, which specifically interact with modified histone proteins [6, 7]. Ever since the initial proposal of the histone code hypothesis [8, 9], much of the effort in chromatin biology has been focused on the identification and mapping of histone PTMs, aiming to understand their functional roles. However, the high number of different histone PTMs raises the question of whether defined combinations of histone PTMs (either on the same tail or within the same nucleosome) carry specific biological meanings beyond the roles of the individual marks [10]. This hypothesis is supported by the fact that many if not all chromatin interacting proteins and complexes bear multiple different binding modules each of them specific for individual PTMs, which allows for combinatorial readout [11–13]. Additionally, chromatin mapping approaches based on correlation and co-occurrence of individual histone PTMs suggested the presence of chromatin states characterized by specific PTM patterns, which are associated with unique functionalities [14, 15]; one example representing the intensively studied H3K4me3–H3K27me3 bivalent state found in ES cells [16, 17].

**Fig. 1:**
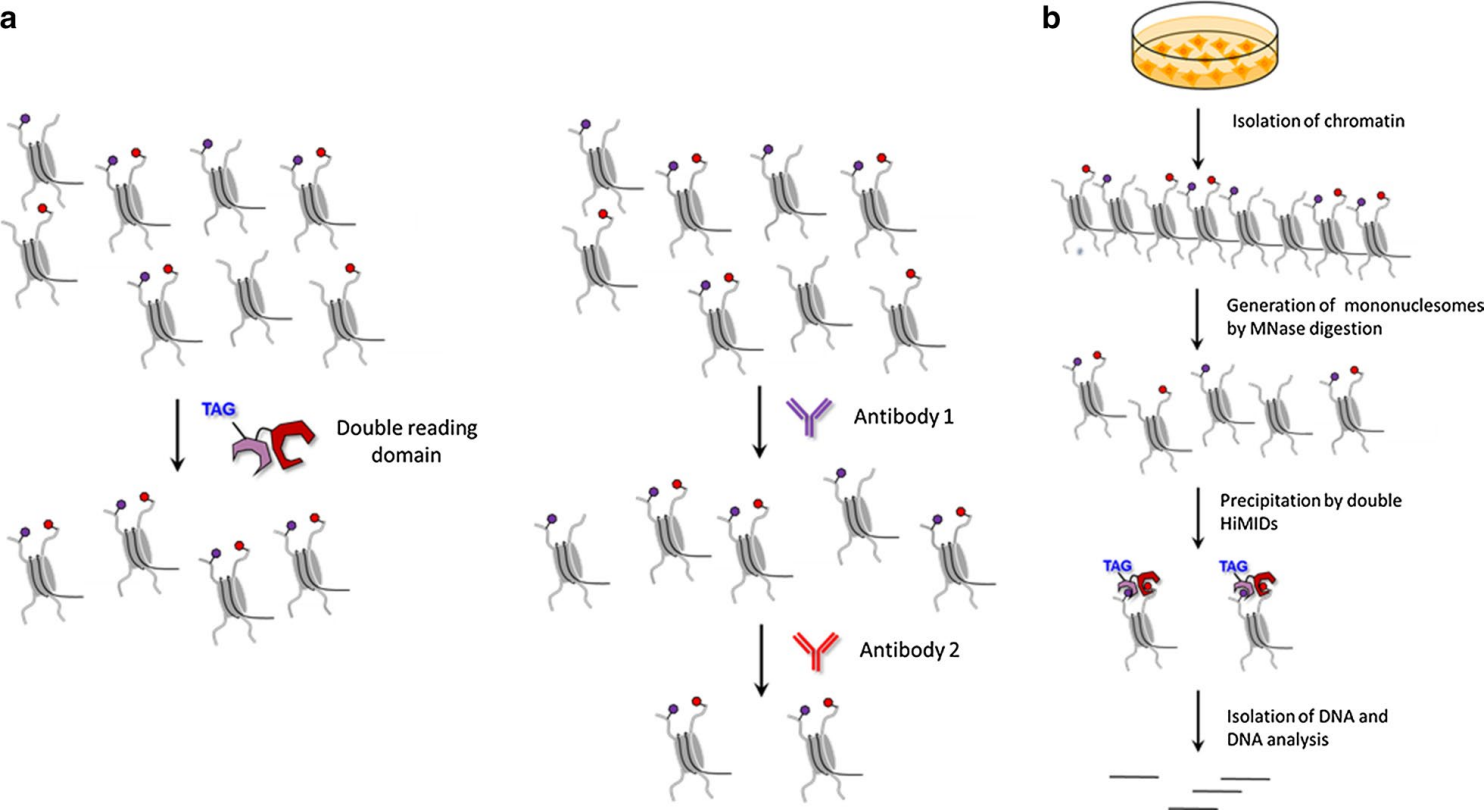
Concepts of this work. **a** Comparison of the novel approach of combined readout of two chromatin marks by double-HiMIDs with the conventional approach of ChIP/re-ChIP, which due to the two consecutive pull-downs typically suffers from low yield. **b** Schematic representation of the procedure of chromatin interacting domain precipitation (CIDOP) experiments using HiMIDs

However, the investigation of the physical co-occurrence of individual histone PTMs on the same histone protein or nucleosome is an experimentally difficult task [13, 18]. The co-occurrence of two or more histone PTMs on a single histone protein can be best studied by top-down or middle-down mass spectrometry [6, 19], but this method cannot detect potential co-occurrence of modifications on different histone tails and it does not allow for association of a particular histone modification with its underlying DNA sequence. Genome mapping of histone PTMs conventionally is conducted by chromatin immunoprecipitation (ChIP) coupled with quantitative PCR (ChIP-qPCR) or massively parallel sequencing (ChIP-seq) using histone PTM-specific antibodies (Fig. 1a). Although such ChIP experiments have made an unparalleled contribution to deciphering the roles of individual histone marks in many nuclear processes, their application in studying the direct coexistence of two or more histone marks has been less straightforward. The reason for this is the high amount of material necessary for two consecutive ChIPs (re-ChIP) and the low amount of recovered DNA, which complicates the preparation of libraries with high quality and complexity suitable for deep sequencing. As a consequence, the vast majority of re-ChIP experiments of histone PTMs so far have been limited to downstream analysis by end-point PCR or quantitative PCR. Most importantly, in conventional ChIP experiments, it remains unclear whether an overlap of individual histone PTMs is due to a true physical coexistence of both marks on one nucleosome or whether they occur separately on adjacent nucleosomes. Moreover, the detection of two marks at one genomic region can be caused by different modification states in different alleles (one allele carrying mark 1 and the second mark 2) or in different cells of a heterogeneous biological sample. To complicate things further, it is difficult to distinguish, whether observed overlaps of histone PTMs merely occur by chance or whether they have specific downstream effects and define unique bivalent (or multivalent) chromatin states. For this, an analysis of the genome-wide distribution of the doubly modified chromatin is necessary; in case of a coincidental co-occurrence, the distribution is expected to reflect the intersection of the distribution of the individual marks, while a distribution showing enrichments in specific target regions can be taken as an indication of a potential functional meaning of the signal.

Methylations of the H3 tail at K9 and K36 are extensively studied and highly conserved histone PTMs. The trimethylation of H3K9 is a hallmark of facultative and constitutive heterochromatin in almost all eukaryotes [20–23], but it is also enriched in silenced genes [24]. By recruitment of reading domains such as the HP1 family members, this modification is involved in gene repression and heterochromatin formation [20]. It is mainly introduced by the SUV39H1, SUV39H2 and SETDB1 enzymes [23], which are essential for embryonic development, heterochromatin formation and gene silencing. H3K36 methylation can be introduced by different protein lysine methyltransferases (PKMTs), but H3K36me3 is exclusively deposited by the SETD2 enzyme, which is targeted by binding to the CTD of the elongating RNAPII [20, 25]. Hence, it follows a gradient of enrichment, starting from the 5′ ends of genes and peaking at their 3′ ends, which is correlated with expression. H3K36me2/3 has a role in the maintenance of repressed chromatin in transcribed regions by recruitment of histone deacetylases [26, 27], H3K4 demethylase LSD2 [28], chromatin remodelers [29] and DNA methylation via the PWWP domain of DNMT3 enzymes [30, 31]. Moreover, the localization of both H3K9me3 and H3K36me2/3 in gene bodies has been associated with alternative splicing [32, 33] and both were found to co-occur frequently in mass spectrometric studies. For example, one study found that about one quarter of H3K9me3 occurs together with H3K36me2/3 on the same H3 tail and, conversely, about one-third of all H3K36me2/3 is paired with H3K9me3 [34] (Additional file 1: Figure S1). In ChIP-seq studies, regions containing H3K9me3 and H3K36me3 dual marks were identified at the 3′ exons of zinc finger (ZNF) genes on chromosome 19 [35] which were later shown to bind ATRX as well [36].

Recently, we have applied recombinant histone modification interacting domains (HiMIDs) as an alternative to histone PTM antibodies in many chromatin biology experiments [37, 38]. Following rigorous quality control criteria, we have shown that selected recombinant HiMIDs perform at least as good as ENCODE validated antibodies [37, 38]. Inspired by the demand for novel reagents necessary for functional analysis of combinatorial chromatin marks, we now extend the HiMID technology by generating artificial double-HiMIDs comprising two well-characterized HiMIDs, fused together in order to exert a combined readout of two histone modifications at the same time in a standard chromatin precipitation experiment (Fig. 1b). Using these novel reagents, we performed ChIP-like experiments, called CIDOP (chromatin interacting domain precipitation) using mononucleosomes prepared from human cells. Doubly modified mononucleosomes were precipitated with the recombinant double-HiMID, and the recovered DNA was analyzed by qPCR and next-generation sequencing. Our data show that properly designed double-HiMIDs enable readout of the coexistence of two histone marks on the same nucleosome. Afterward, we have successfully applied a recombinant double-HiMID designed to bind to H3K9me3 and H3K36me2/3 and discovered that co-occurrence of these two marks defines a new bivalent chromatin state with a distribution that clearly differs from the intersection of the distribution of both individual marks. Our results show that the H3K9me3–H3K36me2/3 state is associated with the bodies of weakly transcribed genes and enhancers, especially of genes involved in cell cycle regulation and metabolism. In addition, we found that it is associated with the zinc finger–Trim28–SetDB1 pathway. Our new reagent allows the direct readout and mapping of two histone modifications physically co-occurring on the same nucleosome, opening the doors for future studies of complex signatures in the histone code.

## Methods

### Cloning, site-directed mutagenesis, expression and purification

The sequences encoding the PWWP domain of murine DNMT3A (279–420 of NP_001258682) and the chromo domain of MPP8 (also known as MPHOSPH8) (57–111 of NP_059990.2) were amplified from existing plasmids [37] and cloned as GST-fusion proteins by Gibson assembly [39] into pGEX-6p-2 vector (GE Healthcare) or pMAL-c2X. Both domains were connected by a natural linker residing between the ADD and PWWP domain of DNMT3A (Additional file 1: Figure S6). The fusion proteins were overexpressed at 18–20 °C in LB medium, induced with 1 mM IPTG at 0.6–0.8 OD_600_ and purified by affinity chromatography as described [40]. The respective mutations (D329A in D3PWWP and F59A in M8Chromo) were introduced by site-directed mutagenesis [41] and validated by restriction analysis and Sanger sequencing.

### Peptide arrays and far-western blot analyses

For far-western blot, native histones were isolated by acid extraction [42] from HEK293 cells. Recombinant histone H3 was purchased from New England Biolabs. Two and a half micrograms of native histones and one microgram of recombinant histone H3 were electrophoresed on a 16–18% SDS-PA gel, transferred on a nitrocellulose membrane and incubated in blocking solution. The binding of recombinant domains to the nitrocellulose membrane and the CelluSpots modified histone tail peptide arrays (Active Motif, Carlsbad, USA) was carried out as described in detail together with the bioinformatic analyses [38, 43]. The full annotation of all spots is provided in Additional file 2: Table S6.

### Antibodies

The following antibodies were used in this study: H3K9me3 (Abcam 8898, lot GR130967-1), H3K36me3 (Abcam 9050, lot GR260274-1), H3K4me3 (Abcam 8580, lot GR85670-1) and H3K27me3 (Active Motif 39155, lot 01613015). Validations of all antibodies are shown in [37, 38].

### Double peptide arrays

Different K9me3 and K36me3 modified and unmodified H3 (1–19) and H3 (26–44) peptides were synthesized by the SPOT method using Fmoc-based chemistry on a MultiPep RSi synthesizer (Intavis AG) and were processed as described in detail previously yielding in solutions of peptides covalently attached to cellulose [44, 45]. From these stock solutions, equal volumes of two peptides were mixed and 50 nl of the mixture spotted with a robust pipetting system on a white-coated glass slide leading to a spot diameter of approximately 500 µm. The binding of the domains to the double peptide spots on the glass slide was tested according to standard western blotting protocols. For blocking, the glass slides were incubated in TTBS with 5% milk and washed two times with TTBS (10 mM Tris–HCl pH 7.5, 0.05% Tween-20, 150 mM NaCl) and one time with interaction buffer (20 mM HEPES pH 7.5, 500 mM KCl, 1 mM EDTA, 0,1 mM DTT, 10% glycerol). Then, the glass slides were incubated for 2 h at RT in 2 ml interaction buffer containing 0.01 µM binding domains. Afterward, the glass slides were washed three times with TTBS and incubated for 1 h at RT with anti-GST AB (1:5000 in TTBS, 1% skim milk, GE Healthcare Life Science 27-4577-01, lot 9541184). After washing three more times with TTBS, the glass slides were incubated for 1 h at RT with horseradish peroxidase conjugated anti-goat AB (1:5000 in TTBS, 1% skim milk, Sigma-Aldrich A4174, lot 071M4767). Finally, the glass slides were washed three times with TTBS and doused with ECL solution (Thermo Fisher Scientific). Images were captured with a FUSION advance solo 4 (PeqLab) under dynamic conditions and analyzed with ImageJ.

### Chromatin precipitation (CIDOP) experiments

The basic CIDOP protocol follows published procedures [37, 38]. It comprises the following steps: preparation of mononucleosomes, pull-down using HiMIDs, isolation of DNA from precipitated nucleosomes and DNA analysis (Fig. 1b). Native mononucleosomes were isolated from HepG2 cells by micrococcal nuclease digestion of intact nuclei obtained as described [46]. Briefly, around 20 million cells were resuspended in 5 ml TM 2 buffer (10 mM Tris–HCl pH7.4, 2 mM MgCl_2_, 0.5 mM PMSF) supplemented with protease inhibitors (cOmplete ULTRA Tablets, Mini, EDTA-free, Easy pack from Roche) and 150 µl of 20% NP-40 and incubated on ice for 5 min. The lysed cells were centrifuged at 1000 rcf for 10 min at 4 °C, and the pellet was washed again with TM2 buffer gently. After a second centrifugation step, the pellet containing the cell nuclei was resuspended in TM2 buffer (200–600 µl depending on the amount of pellet), filled in an Eppendorf tube, pre-warmed at 37 °C and supplemented with 1 mM CaCl_2_. To obtain mononucleosomes, the nuclei were treated with 1.5 µl MNase (New England Biolabs M0247S) per 150 ng of DNA for 5–10 min at 37 °C with pipetting in between gently. The reaction was stopped with 2 mM EGTA. Then, 300 mM of NaCl was added and the sample was centrifuged at 13,000 rcf for 10 min at 4 °C. The supernatant containing the soluble mononucleosomes was collected, snap frozen and kept at − 80 °C until further use.

For pre-clearing, 20 µl of glutathione Sepharose 4B beads or Dynabeads Protein G (Invitrogen) were washed three times with 200 µl DP buffer and incubated with 15–60 µg of the mononucleosomes (based on DNA absorbance) in a final volume of 500 µl DP buffer (16.7 mM Tris–HCl pH8, 167 mM NaCl, 1.1% TritonX-100, 1.2 mM EDTA) supplemented with protease inhibitors (cOmplete ULTRA Tablets, Mini, EDTA-free, Easy pack from Roche) for 1 h at + 4 °C with rotation. Afterward, the supernatant was transferred into a new tube and incubated with 0.5 µM of GST-tagged double-HiMID, H3K9me3 antibody or H3K36me3 antibody overnight at + 4 °C with rotation. The next day, 20–40 µl of pre-washed glutathione Sepharose 4B beads or Dynabeads Protein G (Invitrogen) were added to the supernatant and incubated for 2 h at + 4 °C with rotation. Then, the beads were washed with CIDOP buffers [1 × 1 ml low salt buffer (20 mM Tris–HCl pH 8, 150 mM NaCl, 1% Triton X-100, 2 mM EDTA, 0.1% SDS), 1 × 1 ml high salt buffer (20 mM Tris–HCl pH 8, 500 mM NaCl, 1% Triton X-100, 2 mM EDTA, 0.1% SDS), 1 × 1 ml LiCl buffer (10 mM Tris–HCl pH 8, 250 mM LiCl, 1% Nonident-P40, 1 mM EDTA, 1% sodium deoxycholate)] and 2 × 1 ml TE buffer (10 mM Tris–HCl pH 8, 1 mM EDTA). In the experiments with the DNMT3A PWWP domain, the washing was carried out three times with 1 ml PB200 buffer (50 mM Tris–HCl, 200 mM NaCl, 1 mM EDTA, 0.5% Nonidet P-40, 2 mM DTT) and twice with 1 ml TE buffer. Between each washing step, the beads were incubated for 10 min at + 4 °C with rotation, spun down for 2 min at 2000 rcf and the supernatant discarded.

The bound chromatin was eluted in 100 µl elution buffer (50 mM Tris–HCl, 50 mM NaCl, 1 mM EDTA, 1% SDS) supplemented with 3 µl proteinase K (NEB # P8107S) for 45 min at room temperature with rotation and then incubated at + 55 °C for 60 min to improve the activity. The DNA was recovered using the Chromatin IP DNA Purification Kit from Active Motif.

### Sequential CIDOP/ChIP

The coexistence of H3K9me3 and H3K36me3 was tested by sequential CIDOP/ChIP. For the first pull-down with the H3K9me3-specific M8Chromo HiMID, the CIDOP protocol was used as described above. After the last washing step, the HiMID bound complex was eluted with 200 µl DP buffer containing 40 mM reduced l-glutathione (Applichem) for 2 h at 4 °C with rotation. The supernatant was transferred into a new tube, 20 µl was used for PCR analysis, and the rest was incubated with 3 µl of H3K36me3 antibody overnight at +4 °C with rotation. The next day, 40 µl of pre-washed Dynabeads Protein G (Invitrogen) was added to the supernatant and incubated for 2 h at + 4 °C with rotation. Then, the beads were washed with CIDOP buffers [1 × 1 ml low salt buffer (20 mM Tris–HCl pH 8, 150 mM NaCl, 1% Triton X-100, 2 mM EDTA, 0.1% SDS), 1 × 1 ml high salt buffer (20 mM Tris–HCl pH 8, 500 mM NaCl, 1% Triton X-100, 2 mM EDTA, 0.1% SDS), 1 × 1 ml LiCl buffer (10 mM Tris–HCl pH 8, 250 mM LiCl, 1% Nonident-P40, 1 mM EDTA, 1% sodium deoxycholate)] and twice with 1 ml TE buffer (10 mM Tris–HCl pH 8, 1 mM EDTA). The bound chromatin was eluted in 100 µl elution buffer (50 mM Tris–HCl, 50 mM NaCl, 1 mM EDTA, 1% SDS) supplemented with 3 µl proteinase K (NEB # P8107S) for 45 min at room temperature with rotation and then incubated at + 55 °C for 60 min to improve the activity. The DNA was recovered using the Chromatin IP DNA Purification Kit (Active Motif). The recovered DNA of both steps was analyzed by semiquantitative PCR with different primers to detect the presence of the H3K9me3 and H3K36me3 marks.

### qPCR analysis

The quantitative PCR assays were performed on a CFX96 Real-Time detection system (Bio-Rad) using SsoFast EvaGreen supermix (Bio-Rad). A standard curve was generated to calculate the percent of precipitated DNA and test the efficiency of each primer set. The primer sequences are listed in Additional file 1: Tables S4, S5. Each sample was analyzed in 3 technical replicates. Biological repetition was conducted as indicated in the main text, and error margins were based on biological repeats.

### CIDOP-seq and ChIP-seq

Before proceeding to Illumina sequencing on the HiSeq 2500 platform, the quality of DNA precipitation and library generation was checked with BioAnalyzer (Agilent Technologies, Santa Clara, USA). DNA fragments < 200 bps corresponding to mononucleosomes were isolated from gel and used for library generation. Around 35–60 million, 100-nt sequence reads were obtained and mapped to hg38 with bowtie [47] within the Chipster or Galaxy platform [48, 49] retaining only uniquely mapped reads. Peaks were called with MACS [50] using the broad option and using MACS2 within the Chipster platform. Peak intersection was determined using BEDtools [51]. Density profiles of normalized reads per kilobase per million (RPKM) were generated in DeepTools [52] and visualized in the Integrative Genomics Viewer [53].

For definition of no H3K9me3 and/or H3K36me2/3, H3K9me3-only, H3K36me3-only and overlap of H3K36me3 and H3K9me3 chromatin states, the genome was divided into 1-kb or 3-kb bins and the number of normalized (to the highest dataset) reads per million (RPM) was quantified using the SeqMonk software (http://www.bioinformatics.babraham.ac.uk/projects/seqmonk/).

The distribution of genes per chromosome, peaks and overlap with chromatin segments was determined with EpiExplorer [54], and seqMINER was used for *k*-means clustering and heatmap generation [55]. The Spearman correlation of raw data in 10-kb bins and the metagene profiles were generated in DeepTools [52]. The GO analysis of clusters obtained by *k*-means clustering was carried out in ChIP-Enrich [56]. *P* values for the gene sets were corrected for multiple testing using the Benjamini–Hochberg false discovery rate approach. For GO analyses, the first 10–15 categories termed “biological process” were selected.

The ChIP-seq datasets of H3K4me1, ZNF274, SetDB1 and KAP1 (Trim28) were downloaded from ENCODE [57] and further mapped to Hg38 following our ChIP-seq bioinformatics pipeline. The ZNF274, SetDB1 and Trim28 peaks were downloaded from ENCODE and liftOvered to Hg38 [58].

### RNA-seq data analysis

For RNA-seq, available datasets from HepG2 cells [57] produced by Caltech were used:

wgEncodeCaltechRnaSeqHepg2R1x75dFastqRep1.fastq.gz.

wgEncodeCaltechRnaSeqHepg2R1x75dFastqRep2.fastq.gz.

The reads were mapped with TopHat from the Tuxedo Suite package [59] using default settings. The transcript assembly from both replicates was carried out in the RNA pipeline in SeqMonk, and the transcript list from both replicates was merged. All transcripts were ranked based on RPKM (reads per kilobase of exon per million fragments mapped) and segregated in four groups based on their frequency distribution: no expression, low expression 1, low expression 2, medium expression, high expression.

## Results

Recently, we have shown that the chromodomain from MPP8 (also known as MPHOSPH8) (M8Chromo) and the PWWP domain from DNMT3A (D3PWWP), which bind to H3K9me3 and H3K36me2/3, respectively, can be used as an alternative for histone PTM antibodies [37]. Previous studies have reported the co-occurrence of these marks (Additional file 1: Figure S1) suggesting the potential formation and functional role of the corresponding bivalent chromatin state. In order to directly study the coexistence of H3K9me3 and H3K36me2/3 and map its genome-wide distribution, we sought to extend our HiMID technology, from the readout of single histone PTMs to the readout of two modifications at the same time (Fig. 1). To achieve this, we fused the D3PWWP and the M8Chromo domains using a natural linker in the PWWP domain to generate a hybrid protein with N-terminal GST-tag (GST-D3PWWP-M8Chromo). By doing this, we hypothesized that the divalent interaction with two histone PTMs of the hybrid double-HiMID will be stronger than the sum of the single monovalent interactions due to multidentate binding [11, 60], thus allowing for selective binding of doubly modified nucleosomes.

### Dual readout of H3K36me2/3 and H3K9me3 leads to synergistic binding in vitro

**Fig. 2:**
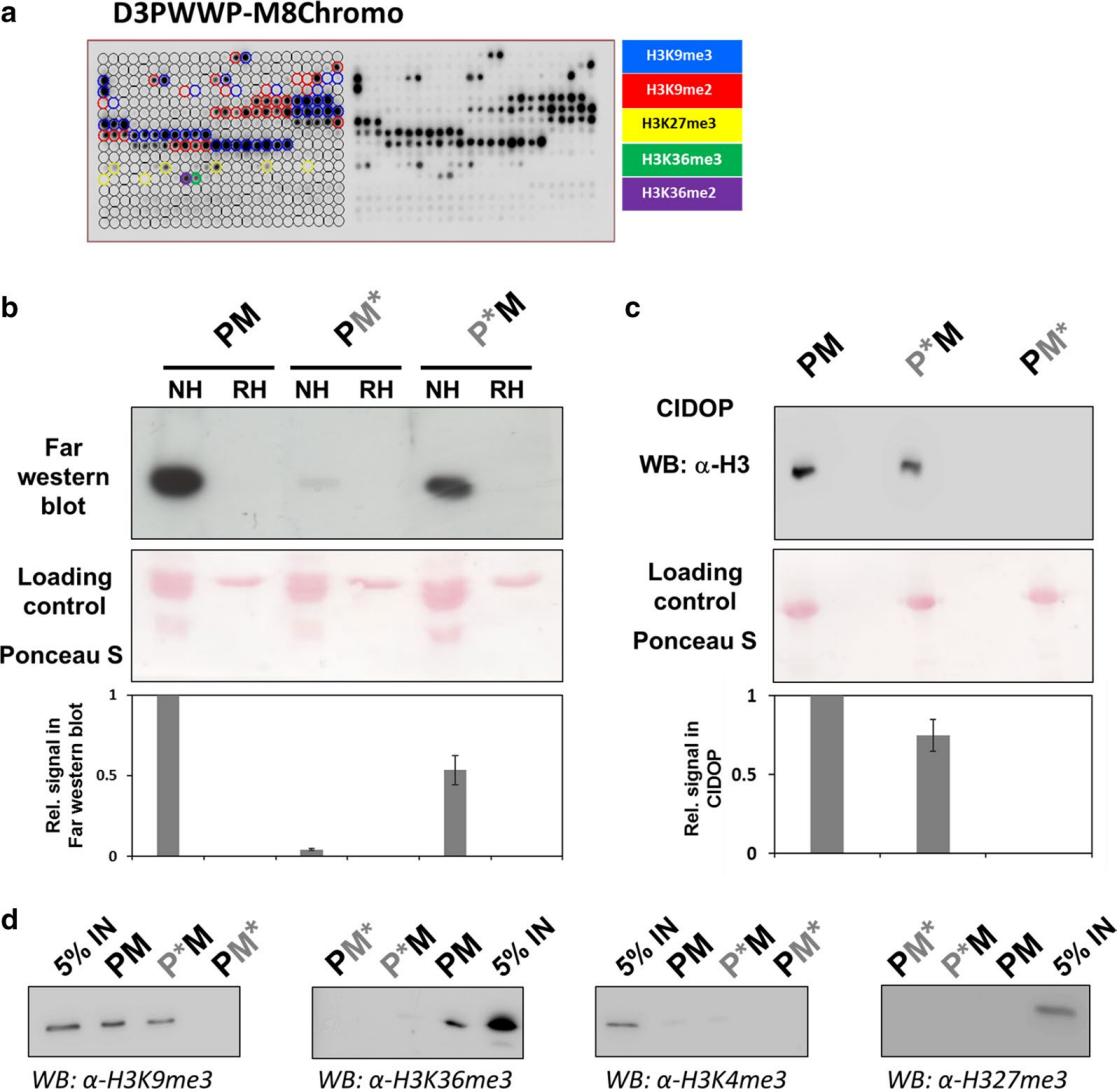
The D3PWWP-M8Chromo double reading domain specifically interacts with native histones and nucleosomes. **a** Peptide array specificity profiling of the hybrid D3PWWP-M8Chromo recombinant protein. The peptide spots are annotated on the left side of the array, and the color code denotes the presence of the designated histone PTM in different combinations. The D3PWWP-M8Chromo domain displayed binding to H3K9me3, H3K27me3, H3K36me2 and H3K36me3 containing peptides. See also Additional file 1: Figure S2A and Additional file 2: Table S6. **b** Far-western blot analyses with D3PWWP-M8Chromo (PM) and its variants containing mutations in the M8Chromo (PM*) or D3PWWP (P*M) domains with native histones (NH) and recombinant histones (RH). The bar diagram shows a quantification of the data based on two repetitions. Error bars indicate the SEM. **c** Precipitation of mononucleosomes with D3PWWP-M8Chromo WT and its variants under stringent (CIDOP) washing conditions and detection with anti-H3 antibody. The Ponceau S staining represents a loading control of D3PWWP-M8Chromo and its variants. The bar diagram shows a quantification of the data based on two repetitions. Error bars indicate the SEM. See also Additional file 1: Figure S2B and C. **d** Specificity of the mononucleosomal CIDOP with D3PWWP-M8Chromo (PM) and its variants containing mutations in the M8Chromo (PM*) or D3PWWP (P*M) domains analyzed by western blot with the H3K9me3, H3K4me3 and H3K27me3 antibodies. 5% IN—5% input. See also Additional file 1: Figure S2

To validate our reagent, first we employed CelluSpots modified histone tail peptide array profiling to confirm that the double-HiMID has retained the two specificities emanating from both individual domains. These arrays contain 384 histone peptides carrying 59 post-translational modifications in different combinations and can be used as cheap and efficient tool to analyze the specificity of histone peptide interaction of reading domains and antibodies [43, 61]. As expected, the D3PWWP-M8Chromo protein bound to peptides harboring H3K9me3, H3K27me3 and H3K36me2/3 (Fig. 2a) [30, 37, 61]. The binding of H3K27me3 peptides by M8Chromo is a peptide binding artifact, which does not occur on full-length histones and nucleosomes [37]. As controls, two protein variants with inactivating binding pocket mutations in one of the domains were used: D3PWWP*-M8Chromo contained a D329A exchange in D3PWWP which inactivates K36me2/3 binding [30] and D3PWWP-M8Chromo* contained an F59A exchange in M8Chromo which inactivates K9me3 binding [37]. As expected, D3PWWP*-M8Chromo only bound to H3K9me3 and D3PWWP-M8Chromo* only bound to H3K36me2/3 (Additional file 1: Figure S2A). These data confirm that both domains in the dual reader recognize their cognate targets.

In order to further validate the specificity of the dual reader, we carried out far-western blot experiments with modified histones isolated from human cells and unmodified, recombinant histones. The D3PWWP-M8Chromo (PM) protein and the corresponding binding pocket variants showed binding to native histones, but not to recombinant histones indicating that they recognize only modified histones (Fig. 2b). In this experiment, the pull-down of native histones by the fully functional D3PWWP-M8Chromo was more efficient than that by the D3PWWP*-M8Chromo (P*M) or D3PWWP-M8Chromo* (PM*) variants, which could be due to better binding of the double active hybrid domain to doubly modified histones or to binding of both types of single modified histones. The very weak binding of the D3PWWP-M8Chromo* protein which only carries an intact PWWP domain is in agreement with our previous findings [37] and with K_d_ values determined for the peptide binding of the isolated domains (M8Chromo 100–200 nM [61, 62], D3PWWP 50–100 µM [30]).

Next, we compared the affinity of the D3PWWP-M8Chromo and variant hybrid proteins in precipitation experiments of mononucleosomes prepared from human cells (Additional file 1: Figure S2B) with stringent washing buffers, typically used in chromatin immunoprecipitation (ChIP) or chromatin interacting domain precipitation (CIDOP) experiments. Precipitated mononucleosomes were detected by western blot with anti-histone H3 antibody (Fig. 2c). While the D3PWWP*-M8Chromo domain showed a robust pull-down, the D3PWWP-M8Chromo* did not pull-down nucleosomes under these conditions, because the PWWP is functional only under less stringent washing conditions [37]. Interestingly, the D3PWWP-M8Chromo double-HiMID was more efficient in nucleosome precipitation than the D3PWWP*-M8Chromo domain (Fig. 2c). These results show that under stringent washing conditions, a larger amount of nucleosomes was precipitated by the D3PWWP-M8Chromo double domain than by the D3PWWP*-M8Chromo single reading domain, which can engage only in monovalent chromatin interactions. This observation suggests that the D3PWWP-M8Chromo double-HiMID binds doubly modified nucleosomes with higher affinity due to bivalent binding, which is particularly striking, as the amount of H3K9me3–H3K36me2/3 double modified mononucleosomes in the input by definition must be smaller than the total amount of mononucleosomes carrying H3K9me3 regardless of the modification state of K36. To confirm that the double-HiMIDs retained the specificities of the single domains in CIDOP reactions as well, the mononucleosomal pull-down reactions were repeated and analyzed by western blot with H3K9me3 and H3K36me3 antibodies for specific binding and with H3K4me3 and H3K27me3 antibodies detecting unrelated marks to assess specificity (Fig. 2d). As observed before, the D3PWWP-M8Chromo* double-HiMID did not show an efficient pull-down under these washing condition. As expected, the H3K9me3 and H3K36me3 modifications were detectable in the D3PWWP-M8Chromo pull-down, H3K9me3 also in the pull-down with the D3PWWP*-M8Chromo double-HiMID. No signal was detectable with the H3K4me3 and H3K27me3 antibodies, demonstrating the specificity of the CIDOP reaction. As before, the H3K9me3 and H3K36me3 signals of the intact double domain were stronger than those of the mutated versions with only one active domain.

**Fig. 3:**
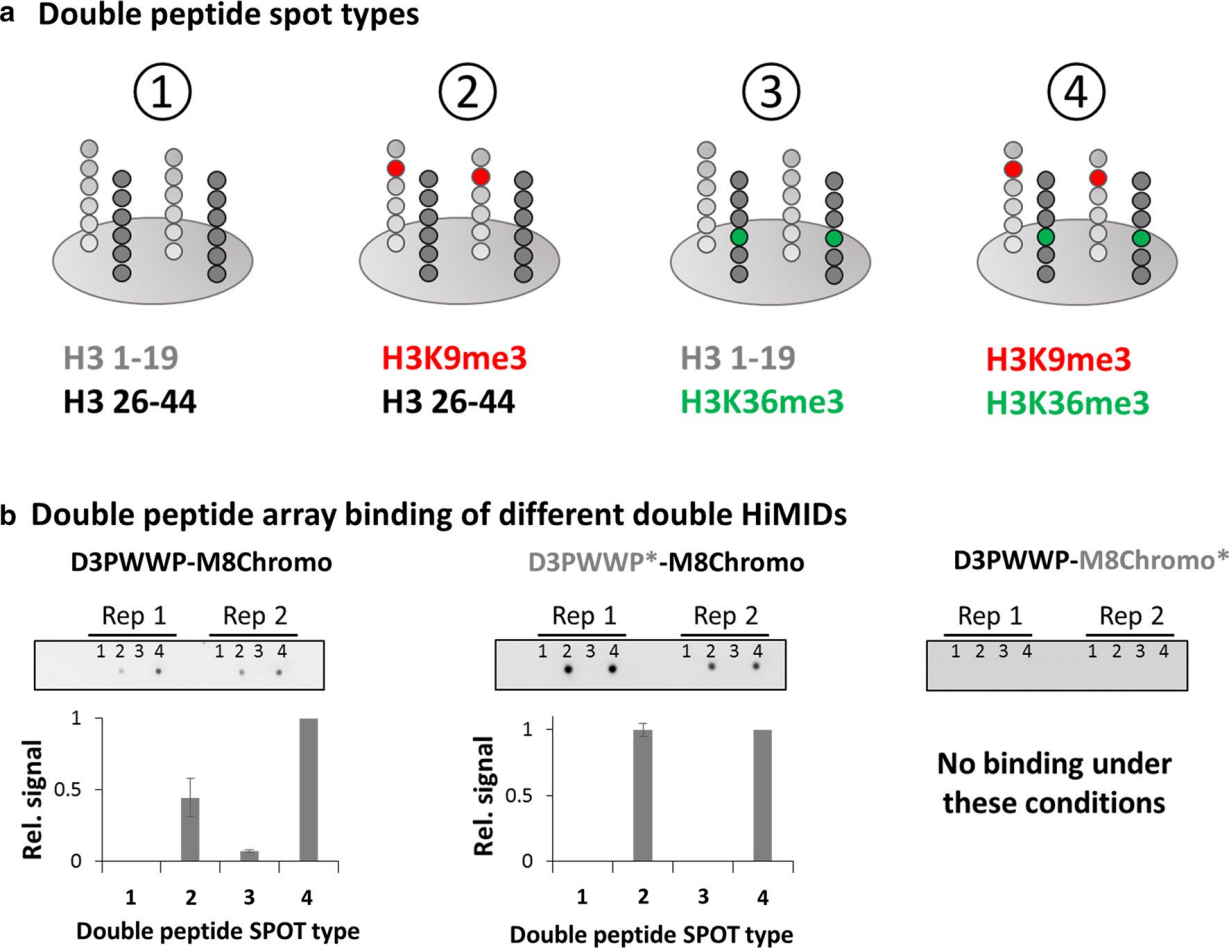
Double peptide array experiments confirm bivalent binding of D3PWWP-M8Chromo to H3K9me3 and H3K36me3. **a** Schematic view of the double peptide arrays. Unmodified, K9me3 and K36me3 modified H3 (1–19) and H3 (26–44) peptides were mixed in all four possible combinations [(*1*) unmodified H3 (1–19) and unmodified H3 (26-44), (*2*) H3K9me3 modified H3 (1–19) and unmodified H3 (26–44), (*3*) unmodified H3 (1–19) and H3K36me3 modified H3 (26–44), and (*4*) H3K9me3 modified H3 (1–19) and H3K36me3 modified H3 (26–44)] and spotted on a glass slide. See also Additional file 1: Figure S3. **b** Binding of the D3PWWP-M8Chromo, D3PWWP*-M8Chromo and D3PWWP-M8Chromo* double-HiMIDS to the double peptide array. The array contains two replicates of the four spots. The bar diagrams show the quantification of the double array based on two repetitions. Error bars indicate the SEM

To investigate the combined interaction of the double-HiMID with two modified peptides, we mixed H3 (1–19) and H3 (26–44) peptides with or without K9me3 or K36me3 modifications in all possible combinations and spotted the mixtures on a glass slide (Fig. 3a). The presence of the H3K9me3 and H3K36me3 marks was validated by antibody binding (Additional file 1: Figure S3). Afterward, the glass slides carrying the double peptide arrays were incubated under stringent high salt conditions with the D3PWWP-M8Chromo double-HiMID and the corresponding binding pocket mutants (Fig. 3b). D3PWWP-M8Chromo showed a preference for binding to the peptide spot containing both modified peptides. In contrast, D3PWWP*-M8Chromo showed an equal binding to H3K9me3 peptides regardless of the modification state of K36 in H3 (26–44). The D3PWWP-M8Chromo* did not bind to the peptides under these conditions similarly as observed before. These results clearly indicate that the double-HiMID can simultaneously interact with H3K9me3 and H3K36me3 modified peptides and this leads to an increased binding.

The interaction of D3PWWP-M8Chromo with double modified chromatin could be either based on the binding of two separate histone tails harboring H3K36me2/3 or H3K9me3 (in *trans*) or to the binding of one H3 tail containing both H3K36me2/3 and H3K9me3 marks (in *cis*) or a combination of both. To discriminate these alternatives, we investigated binding of D3PWWP-M8Chromo to recombinant H3-GST proteins consisting of the first 60 amino acids of H3 containing trimethyllysine [63] analog at position 9 and position 36. The results of pull-down studies with these substrates suggest an interaction in *trans* (Additional file 3). However, this conclusion needs further validation using modified mononucleosomal pull-downs because it is unclear whether trimethyllysine analogs are fully efficient binding substrates for the respective HiMIDs and whether the modified H3-GST protein sufficiently mimics a nucleosome in this assay, which also contains DNA and provides additional protein binding interfaces.

### CIDOP-seq with double-HiMID shows enrichment of bivalent H3K9me3–H3K36me2/3 modified chromatin

**Fig. 4:**
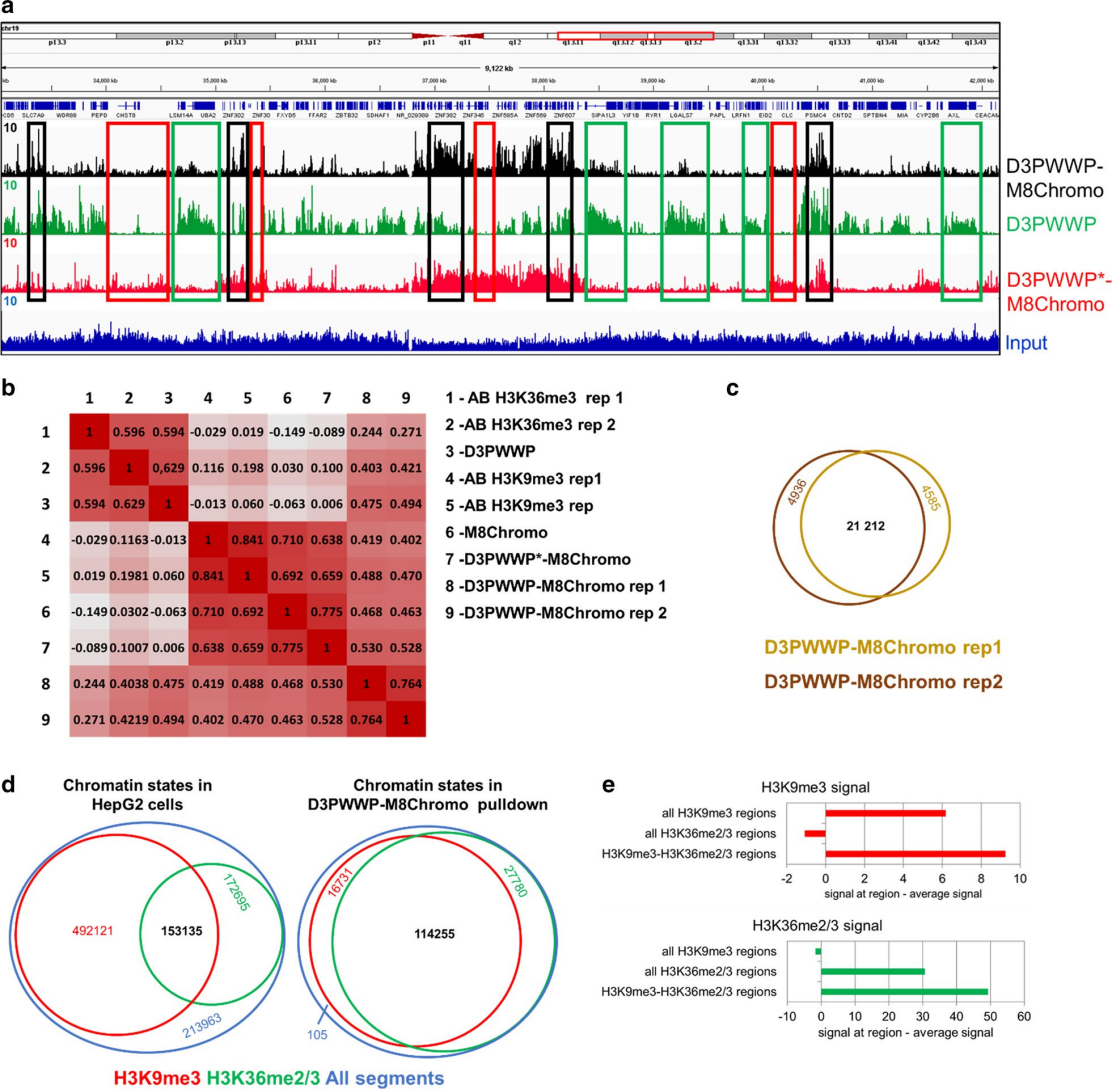
CIDOP-seq with the D3PWWP-M8Chromo double-HiMID. **a** Genome browser view of the CIDOP-seq signal from D3PWWP-M8Chromo and its variants. Note that strong D3PWWP-M8Chromo signal is only observed when DNMT3A PWWP and D3PWWP*-M8Chromo signals are present (black boxes). The green boxes denote examples of the presence of DNMT3A PWWP signal and the absence of D3PWWP*-M8Chromo signal. The red boxes denote examples the presence of D3PWWP*-M8Chromo signal and the absence of DNMT3A PWWP signal. **b** Heatmap of the Spearman correlation coefficients of raw read densities in 10-kb bins of D3PWWP-M8Chromo and D3PWWP*-M8Chromo CIDOP results with D3PWWP CIDOP and various antibody ChIP-seq data taken from Kungulovski et al. [37]. **c** Peak overlap between D3PWWP-M8Chromo replicates 1 and 2. **d** Analysis of chromatin states in HepG2 cells based on H3K9me3 and H3K36me2/3 CIDOP-seq signals using 3-kb bins. The red circle indicates the number of regions with H3K9me3 signal, the green circle indicates regions with H3K36me2/3 signal, and the blue circle indicates all regions. Red numbers refer to only H3K9me3 regions, green number to only H3K36me3 region, black numbers to regions with both signals, blue numbers to regions without both signals. The pull-down by D3PWWP-M8Chromo shows a strong and selective enrichment of the H3K9me3–H3K36me2/3 dual state. See also Additional file 1: Figure S4. **e** Average H3K9me3 and H3K36me2/3 signal intensity determined with the single reading domains at all H3K9me3 regions, all H3K36me2/3 regions and regions carrying the H3K9me3–H3K36me2/3 double mark. In each case, the genome-wide average signal has been subtracted

Since the above biochemical data showed that the double-HiMID interacts with H3K36me3 and H3K9me3 modified H3 tails, we were encouraged to investigate the application of the double-HiMID in chromatin interacting domain precipitation (CIDOP) experiments coupled with qPCR or NGS [37]. In principle, hybrid double domains can precipitate nucleosomes in two modes: either when any of the single or both marks are present (OR-logic) or only when both marks are present at the same time (AND-logic). To discriminate between these modes of action in whole genome analysis approaches, we carried out two CIDOP-seq experiments with D3PWWP-M8Chromo and two experiments with D3PWWP*-M8Chromo, which has an inactivated PWWP domain (Fig. 4a). The D3PWWP-M8Chromo CIDOP-seq replicates were strongly correlated with one another, clustered together (Fig. 4b), and showed a large overlap of annotated peaks (Fig. 4c). Similarly, the D3PWWP*-M8Chromo repeats were highly correlated and they clustered with the MPP8 Chromo single domain and anti-H3K9me3 antibody signals (Fig. 4b). As shown before, we were unable to carry out a successful CIDOP with D3PWWP-M8Chromo* under the washing conditions used here. Therefore, we used the previous DNMT3A PWWP CIDOP-seq data for all further comparative analyses [37] which were obtained under less stringent washing conditions. This dataset represented a third cluster (Fig. 4b) highly correlated with anti-H3K36me3 antibody signals validating its usage as marker for H3K36me2/3. All these three clusters (D3PWWP-M8Chromo, D3PWWP*-M8Chromo and D3PWWP) are only moderately correlated with each other, once more illustrating the clear difference in specificity between D3PWWP-M8Chromo and its constituting single domains. Visual inspection of the data showed that strong D3PWWP-M8Chromo signals were present only in regions where individual H3K9me3 and H3K36me2/3 signals overlapped (Fig. 4a). In contrast, weaker D3PWWP-M8Chromo signals were observed in regions carrying only H3K36me2/3 or only H3K9me3 signals. This result suggests that with an appropriate signal threshold the D3PWWP-M8Chromo double-HiMID can be used as pull-down reagent to directly identify H3K9me3 and H3K36me2/3 doubly modified chromatin, in particular when used along with the corresponding single HiMIDs or domain inactive variants. To corroborate this conclusion, we binned the genome in 3-kb windows and defined four chromatin states based on the distribution of H3K9me3 (merged MPP8 Chromo and D3PWWP*-M8Chromo data) and H3K36me2/3 (DNMT3A PWWP data): (1) without H3K9me3 and H3K36me2/3, (2) H3K36me2/3-only, (3) H3K9me3-only and (4) overlap of H3K36me2/3 and H3K9me3 (Fig. 4d). Interestingly, we observed a very strong enrichment of the bivalent H3K9me3–H3K36me2/3 state in the D3PWWP-M8Chromo pull-down. While overall only 15% of all regions were assigned to the bivalent H3K9me3–H3K36me2/3 state, this fraction increased to 72% in the D3PWWP-M8Chromo pull-down. We observed a 74% recovery of all H3K9me3–H3K36me2/3 overlap regions, while the H3K9me3-only and H3K36me2/3-only regions were recovered much less efficiently (3 and 16%, respectively). Very similar conclusions were drawn from an analysis using 1-kb bins and another analysis using more stringent signal thresholds (Additional file 1: Figure S4).

Next, we were interested to find out whether a more quantitative analysis of the CIDOP signals can discriminate between a model proposing the coexistence of both modifications at H3K9me3–H3K36me2/3 regions, as opposed to a model assuming a mixture of nucleosomes containing either H3K9me3 or H3K36me2/3 that could result from an inhomogeneous sample or allele-specific modification patterns. As first approach, we assumed that a mixture of nucleosomes carrying either H3K9me3 or H3K36me2/3 should result in a lower overall signal for each individual mark in H3K9me3–H3K36me2/3 regions than a sample in which each nucleosome carries both marks. Therefore, we analyzed the average H3K9me3 and H3K36me2/3 signal intensities (taken from the analysis of the single reader or antibodies) at H3K9me3–H3K36me2/3 regions and compared them with the average signals at all H3K9me3 and all H3K36me2/3 regions (Fig. 4e). As expected, we found depletion of H3K9me3 at H3K36me3 regions and vice versa. However, we observed that the individual H3K9me3 signal at H3K9me3–H3K36me2/3 regions is even stronger than the H3K9me3 signal at all H3K9me3 regions. A similar finding was also made for H3K36me3. This result is not compatible with a model assuming that H3K9me3–H3K36me2/3 regions represent a mixture of molecules only containing H3K9me3 or H3K36me3 single marks and is in favor of a model proposing the coexistence of both modifications.

A second strong experimental hint toward the presence of doubly marked chromatin is the enrichment of H3K36me3 containing nucleosomes by the D3PWWP-M8Chromo double domain that was observed under stringent washing conditions. Under these conditions, no productive interaction of the single D3PWWP subdomain with nucleosomes was possible. Hence, it is likely that the mode of binding is the following: M8Chromo docks to H3K9me3 containing nucleosomes. The presence of H3K36me2/3, which is then bound by D3PWWP, enhances the binding affinity and leads to preference for doubly modified chromatin, rather than H3K9me3-only chromatin.

### Validation of selected H3K9me3–H3K36me2/3 regions with quantitative CIDOP, ChiP and sequential CIDOP–ChIP

**Fig. 5:**
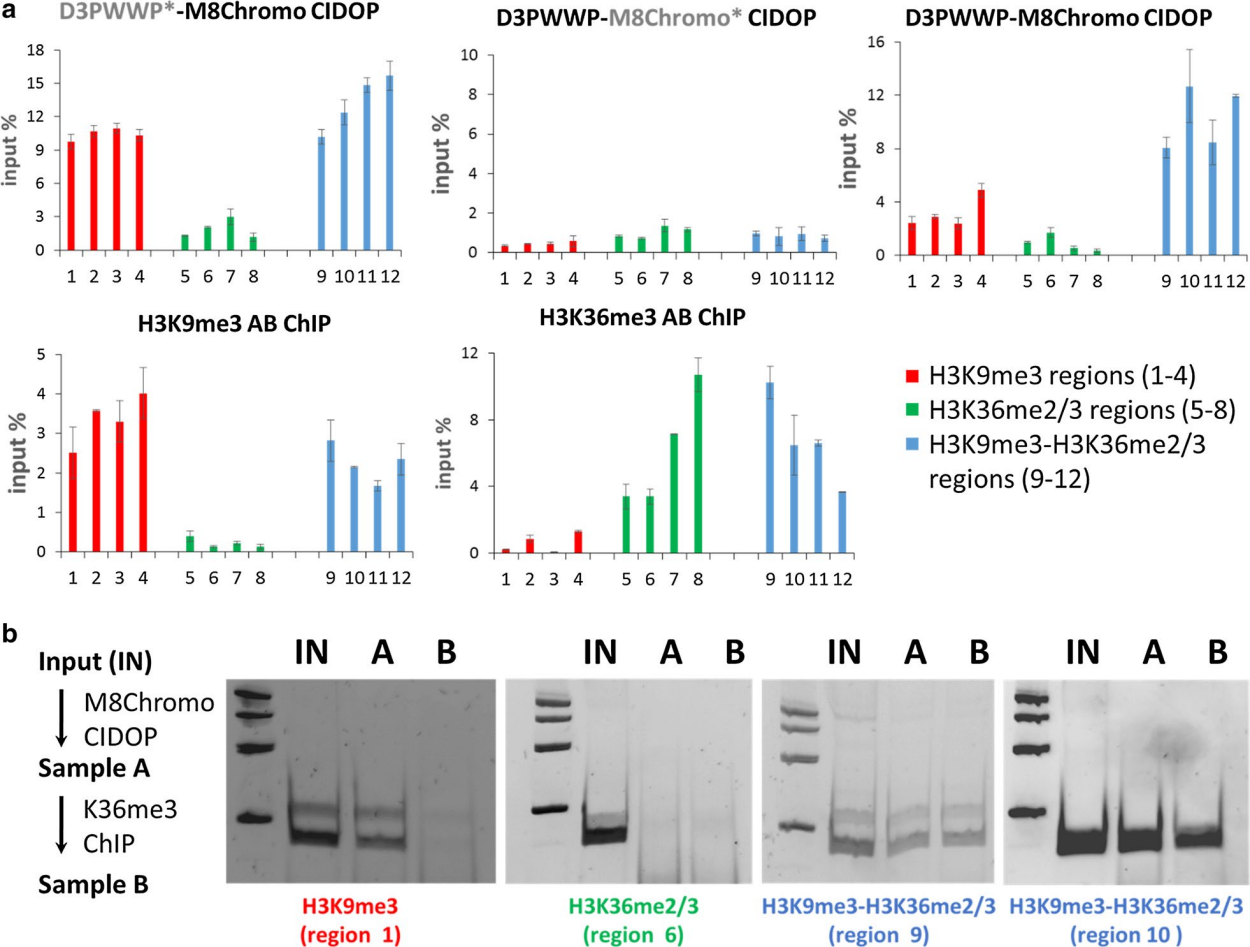
Additional validation of the detection of H3K9me3-H3K36me2/3 double modified chromatin. **a** Chromatin interacting domain precipitation (CIDOP) and ChIP quantified by real-time PCR (qPCR). Twelve regions (Additional file 1: Table S4) containing only H3K9me3, only H3K36me3 and both marks were selected. CIDOP was conducted with the D3PWWP-M8Chromo double domain and the two variants with one inactivated binding pocket D3PWWP-M8Chromo* (with inactivated H3K9me3 binding pocket) and D3PWWP*-M8Chromo (with inactivated H3K36me2/3 binding pocket). In addition, ChIP experiments with H3K9me3 and H3K36me3 antibodies were performed to confirm the presence or absence of both marks. Each experiment was conducted in two biological replicates. Error bars show the SEM. **b** Sequential CIDOP-ChIP experiments were conducted on H3K9me3 only, H3K36me3 only and double mark regions. First, H3K9me3-specific M8Chromo CIDOP was conducted followed by H3K36me3 ChIP. The pull-down was analyzed by semiquantitative PCR

Twelve regions of different modification states were selected for further technical validation in independent CIDOP-qPCR and ChIP-qPCR experiments. Amplicons covering H3K36me2/3 and H3K9me3 CIDOP-seq peak overlap regions were labeled as H3K9me3–H3K36me2/3, while singly modified regions were labeled as H3K36me2/3 or H3K9me3, respectively. Our CIDOP-qPCR data showed binding of the D3PWWP-M8Chromo double domain to all H3K9me3 and H3K36me2/3 regions. However, clear preferences for binding to H3K9me3–H3K36me3 doubly modified regions were observed. In contrast, the mutated D3PWWP*-M8Chromo protein bound to H3K9me3 regions, not discriminating between the absence and presence of the second mark (Fig. 5a). The CIDOP with the D3PWWP-M8Chromo* protein (under less stringent conditions) was weak, but also showed preferences for H3K36me3 containing regions irrespective of the presence of H3K9me3. These results were highly reproducible in biological repetitions of this experiment. In addition, these regions were analyzed with anti-H3K9me3 and anti-H3K36me3 antibodies showing excellent agreement with the CIDOP-seq results. When combined, these data clearly show that strong D3PWWP-M8Chromo signals are indicative of the coexistence of H3K9me3 and H3K36me2/3, while weaker D3PWWP-M8Chromo signals indicate the presence of single modifications.

To further validate the presence of H3K9me3–H3K36me2/3 doubly marked chromatin, we performed sequential CIDOP-ChIP, in which H3K9me3-specific M8Chromo CIDOP was followed by H3K36me3 ChIP using regions containing only H3K9me3, only H3K36me3 or both marks. As shown in Fig. 5b, sequential pull-down was only possible at doubly marked regions, confirming the coexistence of both marks at these sites. All these results clearly confirm that we indeed enriched nucleosomes carrying both marks and not a mixture of nucleosomes in which some carry H3K9me3 and others H3K36me2/3.

### The H3K9me3–H3K36me2/3 bivalent state is enriched in weakly transcribed chromatin

**Fig. 6:**
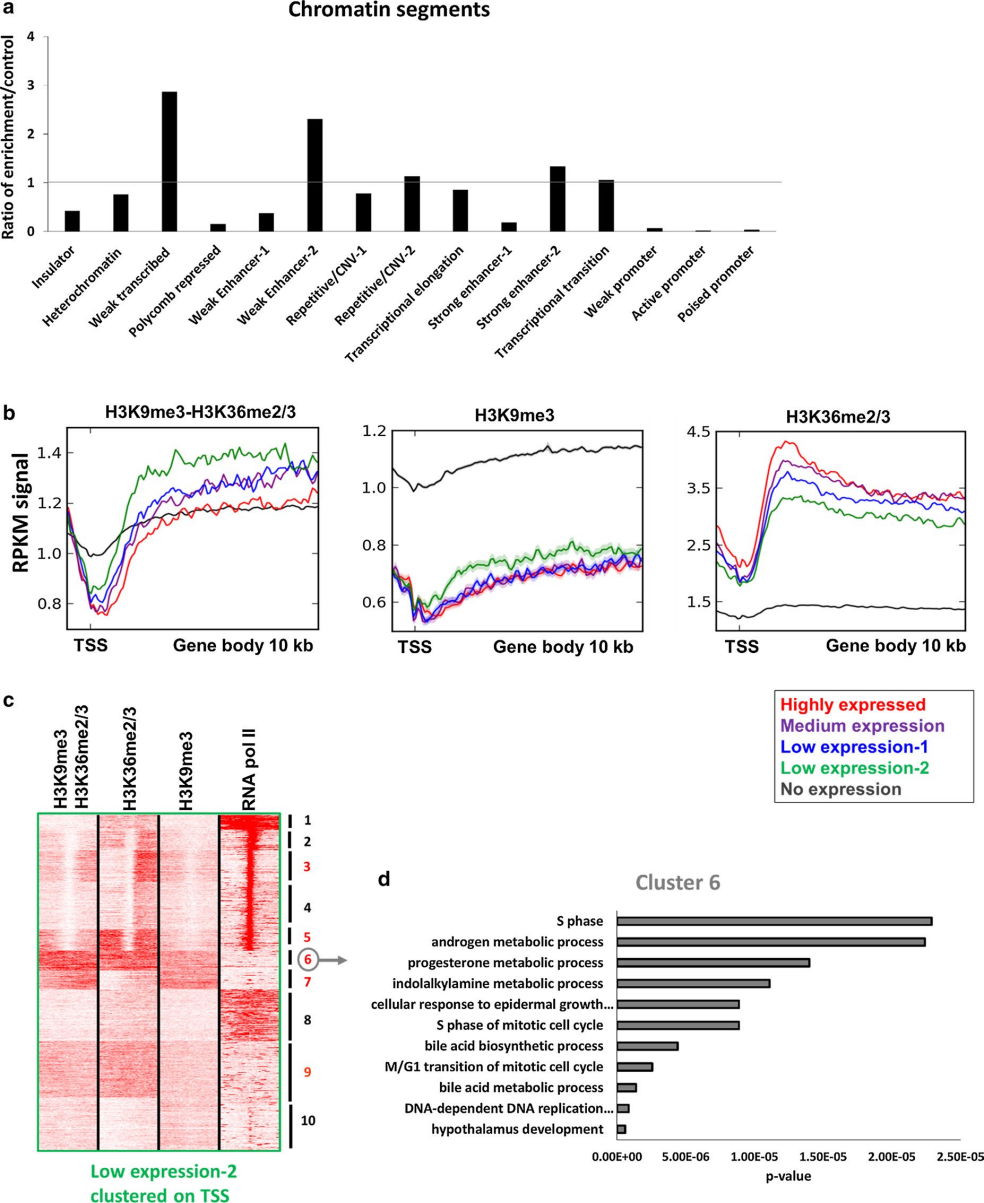
Association of the H3K9me3–H3K36me2/3 bivalent state with genomic states. **a** Overlap of the H3K9me3–H3K36me2/3 bivalent chromatin state with chromatin states defined by Ernst et al. [15]. **b** Metagene analyses of H3K9me3–H3K36me2/3, H3K9me3 and H3K36me2/3 distributions over gene bodies in relation to gene expression. **c** Clustering analysis of tag densities of H3K9me3–H3K36me2/3, H3K9me3, H3K36me2/3 and RNA pol II. Tags were collected in a 10-kb window (− 5 to + 5 kb) centered on the midpoints of TSS from genes with low expression 2 and sorted by *k*-means clustering (ten clusters). Five clusters with clear H3K9me3–H3K36me2/3 signal are annotated with red numbers. Other clusters not showing strong H3K9me3–H3K36me2/3 signal are annotated with black numbers. The image also shows that the H3K9me3–H3K36me2/3 signal is only observed at regions with H3K9me3 and H3K36me2/3 signals confirming the double specificity of the pull-down. **d** Representative gene ontology (GO) analysis of biological processes linked with cluster 6. For more information about all the other clusters, refer to Additional file 1: Table S1

All the data presented in the previous sections indicate that the hybrid affinity reagent made of the DNMT3A PWWP and MPP8 chromodomain specifically recognize doubly modified nucleosomes. For that reason, from now on we refer to the D3PWWP-M8Chromo signal as bivalent H3K9me3–H3K36me2/3 state. Next, we wanted to investigate the distribution of nucleosomes with bivalent H3K9me3–H3K36me2/3 marks in HepG2 cells and check out whether it is non-random (i.e., enriched in defined functional elements), which would suggest that the bivalent H3K9me3–H3K36me2/3 modification represents a chromatin state with a biological role. To this end, we merged the peaks obtained from both CIDOP-seq replicates (Fig. 4c) and used the EpiExplorer database [54] for initial data mining. By comparing the distribution of H3K9me3–H3K36me2/3 peaks and using the same number of randomized peaks as control, we observed an enrichment of H3K9me3–H3K36me2/3 in distinct chromatin states as defined by Ernst et al. [15] (Additional file 1: Figure S5), all of them characterized by weak transcription (Fig. 6a).

### The H3K9me3–H3K36me2/3 bivalent state is enriched in weakly transcribed genes

To validate this observation, we plotted the H3K9me3–H3K36me2/3, H3K9me3 and H3K36me2/3 signals over the promoters and bodies of all genes binned by their expression levels and observed a strong preference of the H3K9me3–H3K36me2/3 bivalent state for genes with low expression (green lines in Fig. 6b), while the distribution of signal over highly expressed and non-expressed genes was similar to each other and lower than over lowly expressed genes (red and black lines). This was in sharp contrast to the individual H3K9me3 and H3K36me2/3 signals indicating the distribution of H3K9me3–H3K36me2/3 is distinct from the random intersection of both individual marks. As expected, H3K9me3 was highly enriched in non-expressed genes, but not in genes with high, medium and low expression, and H3K36me2/3 was enriched in expressed genes (correlated with levels of expression), but not in non-expressed genes (Fig. 6b). To further dissect the chromatin state of genes with the lowest expression (low expression 2), we performed k-means clustering and re-affirmed that indeed several of the obtained clusters exhibit H3K9me3–H3K36me2/3 enrichment (Fig. 6c). Comparison of the clusters confirmed that H3K9me3–H3K36me2/3 signal was only detected if both individual signals were present. The strongest H3K9me3–H3K36me2/3 signal was found in clusters 3, 5, 6, 7 and 9, which encode for gene ontology (GO) categories associated with biological processes such as cell cycle transition, metabolism of nucleotides, morphogenesis and development (especially of bones) and hormone metabolism (Fig. 6d and Additional file 1: Table S1).

### The H3K9me3–H3K36me2/3 bivalent state is enriched in particular subtypes of enhancers

**Fig. 7:**
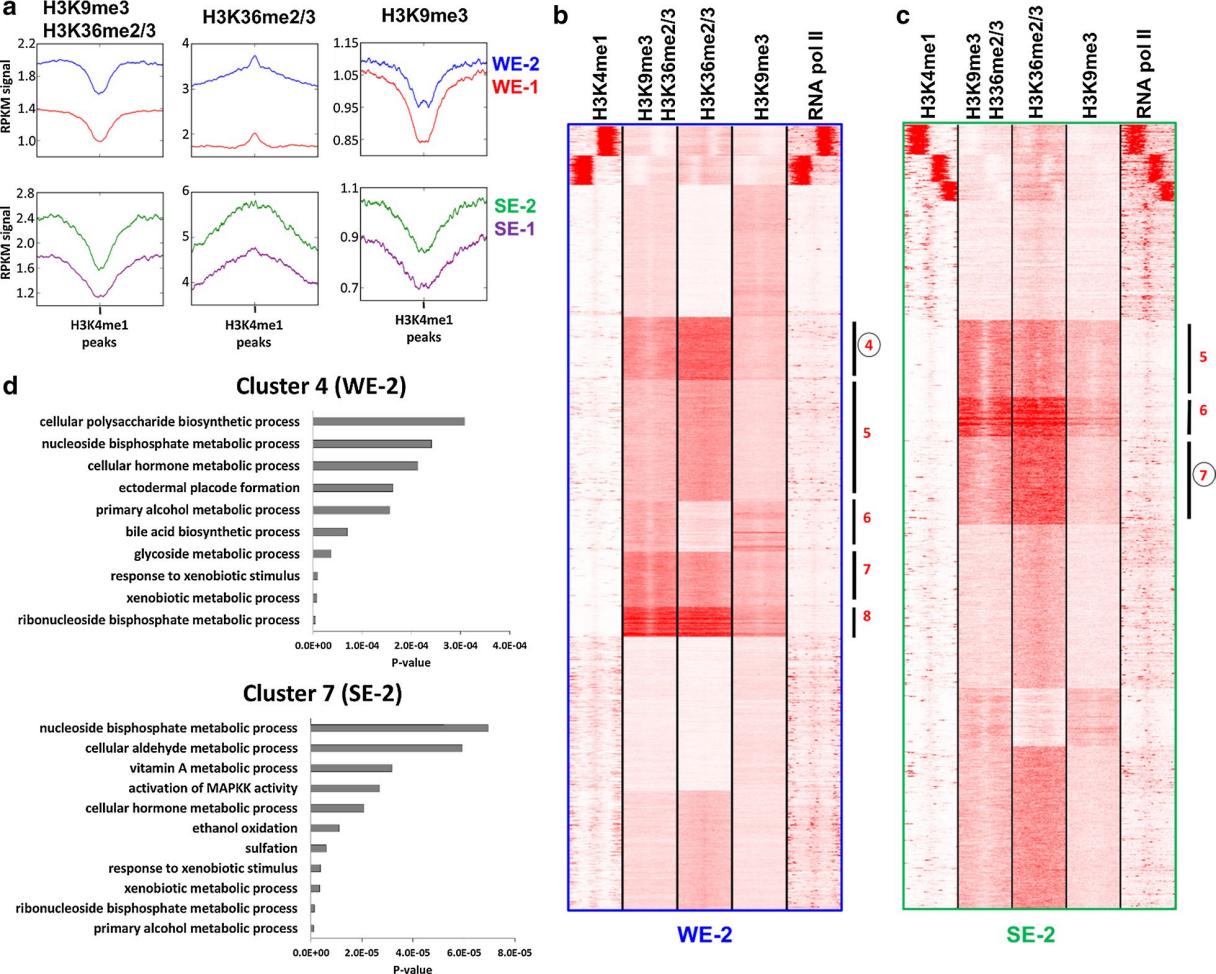
Association of the H3K9me3–H3K36me2/3 bivalent state with enhancer types. **a** Composite profiles of H3K9me3–H3K36me2/3, H3K9me3 and H3K36me2/3 distribution, centered on H3K4me1 peaks associated with the indicated types of enhancers. **b**, **c** Clustering analysis of tag densities of H3K9me3–H3K36me2/3, H3K9me3, H3K36me2/3 and RNA pol II. Tags were collected in 10-kb windows (− 5 to + 5 kb) centered on the midpoints H3K4me1 peaks associated with the chromatin states WE-2 (**b**) or SE-2 (**c**) and sorted by *k*-means clustering (ten clusters). Clusters with clear H3K9me3–H3K36me2/3 signal are labeled with red numbers. Other clusters not showing strong H3K9me3–H3K36me2/3 signal are not annotated. The image also shows that H3K9me3–H3K36me2/3 signal is only observed at regions with H3K9me3 and H3K36me2/3 signals confirming AND-readout. **d** Representative gene ontology (GO) analysis of biological processes linked with cluster 4 from WE-2 and cluster 7 from SE-2. For more information about all the other clusters, refer to Additional file 1: Tables S2 and S3

In addition to “weak transcribed” chromatin segments and genes, we also observed an enrichment of the H3K9me3–H3K36me2/3 bivalent state in certain subtypes of enhancers, decorated with H3K4me1 (Fig. 6a). To verify this observation, we selected H3K4me1 ChIP-seq peaks associated with WE-1, WE-2, SE-1 and SE-2 and plotted their H3K9me3–H3K36me2/3, H3K9me3 and H3K36me3 signals (Fig. 7a). In agreement with our previous observations, we detected stronger enrichment of H3K9me3–H3K36me2/3 in WE-2 and SE-2 in comparison with WE-1 and SE-1, respectively. The stronger enrichment of bivalent marks in SE-2 compared to SE-1 is in agreement with the selective enrichment of H3K9ac in SE-1 which competes with H3K9me3. The corresponding single marks were enriched as well, which is in agreement with a previous report [64]. To functionally dissect these two subtypes of enhancers, we performed *k*-means clustering, centered around the midpoints of H3K4me1 peaks associated with WE-2 or SE-2, respectively, and found five clusters (out of ten) with high enrichment of H3K9me3–H3K36me2/3 in WE-2 and 3 clusters (out of ten) in SE-2 (Fig. 7b, c). Then, we linked the H3K4me1 peaks from each cluster to the closest TSS in a distance of at least 10 kb (to associate enhancers with putative target genes but exclude promoters) and carried out GO analyses with these genes. The WE-2 enhancers (cluster 4–8) were associated with genes involved in biological processes such as metabolism of xenobiotics, alcohols, vitamins, lipids and nucleotides, regulation of microtubules, cell cycle and regulation of collagen (Fig. 7d and Additional file 1: Table S2). The SE-2 enhancers (clusters 5–7) were associated with genes involved in biological processes such as regulation of protein localization and transport, skeletal, cartilage and connective tissue morphogenesis and metabolism of xenobiotics, alcohols, lipids, vitamins and nucleotides (Fig. 7d and Additional file 1: Table S3).

### The H3K9me3–H3K36me2/3 bivalent state is associated with the zinc finger–Trim28–SetDB1 pathway

**Fig. 8:**
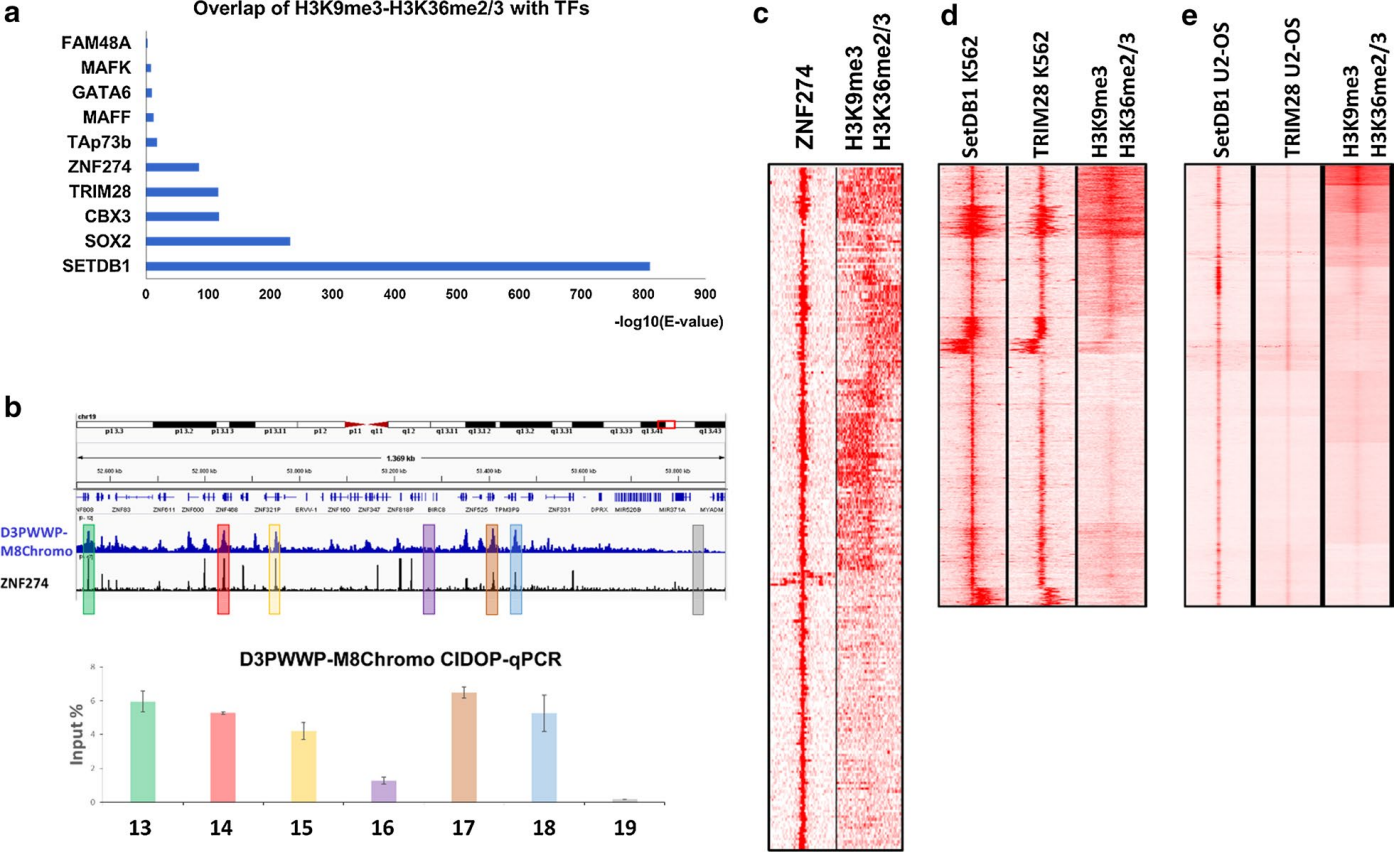
Association of the H3K9me3–H3K36me2/3 bivalent state with the zinc finger–Trim28–SetDB1 pathway. **a** Overlap of H3K9me3–H3K36me2/3 with peaks of DNA-binding factors from the Re-Map database. **b** Genome browser view of a part of chromosome 19 with many zinc finger (ZNF) genes showing co-localization of ZNF274 signal and H3K9me3–H3K36me2/3. D3PWWP-M8Chromo CIDOP-seq data were confirmed by CIDOP-qPCR using 5 H3K9me3–H3K36me2/3 peak and 2 control regions (Additional file 1: Table S5). The error bars show SEM values based on two independent biological repeats. **c** Clustering analysis of tag densities of H3K9me3–H3K36me2/3 and ZNF274. Tags were collected in 20-kb window (− 10 to + 10 kb) centered on the midpoints ZNF274 peaks and sorted by *k*-means clustering (ten clusters). **d** Clustering analysis of tag densities of SetDB1 and TRIM28 from K652 cells with H3K9me3–H3K36me2/3 from HepG2 cells. Tags were collected in 10-kb windows (− 5 to + 5 kb) centered on the midpoints SetDB1 peaks and sorted by *k*-means clustering (ten clusters). **e** Clustering analysis of tag densities of SetDB1 and TRIM28 from U2-OS cells with H3K9me3–H3K36me2/3 from HepG2 cells. Tags were collected in 10-kb windows (− 5 to + 5 kb) centered on the midpoints SetDB1 peaks and sorted by *k*-means clustering (ten clusters)

So far, our data have indicated that the bivalent H3K9me3–H3K36me2/3 chromatin state is associated with weakly expressed genes, which are regulated in a cell type-dependent manner. Therefore, we were interested to investigate whether these regions contain binding sites for regulatory factors. We used the Re-Map database [65] to search for overlap of H3K9me3–H3K36me2/3 peaks with the binding sites of DNA-interacting factors in tens of cell types from hundreds of ChIP-seq experiments. Interestingly, we noticed a very significant overlap of the H3K9me3–H3K36me2/3 bivalent state with binding sites of ZNF274, Trim28, CBX3 and SetDB1, all of which are members of the zinc finger–Trim28–SetDB1 pathway (Fig. 8a). To explore this further, we searched for available ChIP-seq datasets of any of these proteins in HepG2 cells and found one for ZNF274. ZNF274 peaks showed a striking correlation with H3K9me3–H3K36me2/3 signal (Fig. 8b). These regions include ZNF genes on chromosome 19 which have already been described to contain H3K9me3–H3K36me3 marks clustered at the 3′UTR exons [35]. The H3K9me3–H3K36me2/3 signal of representative regions was confirmed by independent CIDOP-qPCR experiments. Clustering of H3K9me3–H3K36me2/3 and ZNF274 signals showed that overall around 60% of all ZNF274 sites are overlaid with H3K9me3–H3K36me2/3 in HepG2 cells (Fig. 8c). Since no SetDB1 and TRIM28 ChIP-seq datasets were available from HepG2 cells, we reasoned that some of their binding sites might be partially conserved between different cell lines and collected SetDB1 and TRIM28 data from K562 and U2-OS cells. Clustering of these data around SetDB1 peaks revealed clusters clearly enriched with H3K9me3–H3K36me2/3 (Fig. 8d, e). These data collectively indicate a link between zinc finger–TRIM28–SetDB1 pathway and the H3K9me3–H3K36me2/3 bivalent chromatin state.

## Discussion

In this study, we developed an innovative strategy for a combined readout of two histone PTMs in a single-step chromatin precipitation using amounts of chromatin that are typically needed in conventional ChIP experiments. This is an extension of the technology that applies recombinant HiMIDs for readout of single histone PTMs in ChIP-like experiments [37, 38]. We showed that the recombinant double-HiMIDs robustly perform in CIDOP-seq experiments. The recombinant nature of these affinity reagents permits for straightforward protein engineering enabling the fusion of individual HiMIDs to generate a double-HiMID, an approach not possible with conventional antibody technology. We show that two well-characterized HiMIDs targeted toward H3K9me3 and H3K36me2/3 after fusion preferentially interact with mononucleosomes bearing both modifications. Systematic repetitions of our CIDOP-qPCR and CIDOP-seq experiments confirmed high reproducibility of the data, indicating that using appropriate signal thresholds (ideally in combination with single HiMIDs or antibodies detecting the individual modifications) double-HiMIDs can be used to directly map doubly modified chromatin, an experimental challenge that cannot be solved easily with existing technologies.

After finishing this work, two alternative approaches for the analysis of double chromatin marks were presented highlighting the importance of this scientific question. Weiner et al. [66] used sequential-ChIP coupled with barcoding, which allowed them to carry out genome-wide studies of H3K4me3 and H3K27me3, as well as H3K9me1 and H3K27ac. In addition, Shema et al. [67] used single-molecule detection of surface immobilized nucleosomes with different antibodies followed by sequencing of the nucleosomal DNA to map combinatorial chromatin modifications. However, both these methods have their limitations as well. The sequential-ChIP procedure contains many steps, and like our method it depends on careful validation of the results. The single-nucleosome approach currently is limited by the throughput, and it does not reach genome-wide coverage.

In the future, many more combinations of HiMIDs can be fused together, validated and applied in studies of other bivalent states or co-occurring histone PTMs such as H3K4me3 and H3K27me3 or H3K9me3 and H4K20me3. The art of the design of double-HiMIDs will be to generate parts in which isolated HiMIDs would not be able to pull-down chromatin under the experimental conditions, but only the synergistic interaction of the double-HiMID bound to both target marks would be sufficiently strong for pull-down. This goal was ideally reached in the case of the D3PWWP domain used here, not yet for M8Chromo. Additional design cycles may allow to change this and further increase the specificity toward doubly modified chromatin. In addition, the PWWP domain of DNMT3A and its DNMT3B paralog [68–70] have been shown to bind DNA which could influence the pull-down efficiency and specificity and needs to be investigated further, for example, using PWWP mutants with lost DNA binding but intact binding of methylated H3K36. As described in the results section, the results with double trimethyllysine analog containing peptides suggest that the D3PWWP-M8Chromo double-HiMID does not interact with single histones containing both modifications. This preliminary finding points into another important direction of further design efforts, because a double-HiMID with cis-binding would be of great applicative value.

We used the newly developed affinity reagent to investigate the localization and distribution of the H3K9me3–H3K36me2/3 bivalent state on a genome-wide scale. We observed a unique distribution of H3K9me3–H3K36me2/3 doubly modified nucleosomes, which is distinct from the distribution of both individual modifications, indicating that the combined deposition of both marks did not occur by chance, but the H3K9me3–H3K36me2/3 signal represents a newly defined bivalent chromatin state. So far, the functional relevance of the co-occurrence of H3K9me3 and K36me2/3 methylations has been elucidated in *S. pombe* only, where it was shown that RNAPII transcribes centromeric repeats in a brief period during the S-phase. Transcription allows for H3K36me2/3 deposition at these sites, which in turn contributes to silencing and heterochromatin formation in a pathway parallel to the Clr4 pathway, which introduces H3K9me3 [71]. The major insight provided in our work is the association of the H3K9me3–H3K36me2/3 bivalent state with weakly transcribed chromatin states such as lowly expressed genes and enhancers. The bivalent H3K9me3–H3K36me2/3 state holds the middle ground between repression and activation, in accord with countless studies, which have reported the association of H3K9me3 with repressed chromatin and H3K36me2/3 with actively transcribed chromatin, due to its deposition by the elongating RNAPII. Our results are in agreement with previous findings of co-occurrence of H3K9me3 and H3K36me2/3 in gene bodies with roles in alternative splicing [32, 33].

We uncovered that the genes associated with these weakly transcribed states in HepG2 cells marked by H3K9me3–H3K36me2/3 have a role in cell cycle regulation, metabolism and signaling. We observed a very prominent co-localization of H3K9me3–H3K36me2/3 with ZNF274 and SetDB1 binding sites (albeit using data from different cell types), indicating that the SetDB1 pathway may intersect with the H3K9me3–H3K36me2/3 chromatin state. In agreement with these findings, zinc finger gene 3′ exons which represent one group of ZNF274 and SETDB1 binding sites were previously shown to contain H3K9me3 and H3K36me2/3 modifications [35, 36]. Interestingly, Blahnik et al. [35] could not find indications for an allelic separation of both marks, suggesting that the regions are bivalently marked as suggested by our data as well. Mechanistically, the connection of the H3K9me3–H3K36me2/3 state to SETDB1 is striking, because this enzyme is one of the main factors introducing H3K9me3. Further studies should additionally dissect the relationship of the coexistence of the bivalent H3K9me3–H3K36me2/3 state and SetDB1 in terminally differentiated and embryonic stem cells.

## Conclusions

We have introduced the application of double-HiMIDs for the direct single-step genome-wide analysis of combinatorial chromatin modifications and validated it using a double HiMID consisting of the MPP8 chromodomain and DNMT3A PWWP domain reading H3K9me3–H3K36me2/3. The application of this and related double-HiMIDs has the potential to propel chromatin research by allowing holistic studies of co-occurrence and distribution of many combinations of chromatin marks in unprecedented detail. This experimental approach will aid the community in providing a novel and very potent method to investigate the combinatorial pattern of histone modifications. Our discovery of a novel H3K9me3–H3K36me2/3 bivalent chromatin state illustrates the power of this approach, and it will stimulate numerous follow-up studies on its biological functions.

## Additional files

**Additional file 1: Figure S1**. Analysis of the coexistence of H3K9me3 and H3K36me2/3 in one H3 molecule by mass spectrometry. **Figure S2.** Control experiments related to Fig. 2. **Figure S3.** Quality control of double peptide spot arrays. **Figure S4.** Definition of chromatin states in HepG2 cells based on H3K9me3 and H3K36me2/3 CIDOP-seq signals using 1 kb bins. **Figure S5.** Definition of chromatin states based on Ernst et al. 2011. **Figure S6.** Annotated sequence of the D3PWWP-M8Chromo double domain. **Table S1.** GO analysis of low expression gene clusters shown in Fig. 6c. **Table S2.** GO analysis of the clusters of genes regulated by weak enhancers-2 shown in Fig. 7b. **Table S3.** GO analysis of the clusters of genes regulated by strong enhancers-2 shown in Fig. 7c. **Table S4.** Sequences of primers used in Fig. 5 for CIDOP-qPCR. **Table S5.** Sequences of primers used in Fig. 8b for CIDOP-qPCR.

**Additional file 2: Table S6.** Full annotation of the peptides present on the CelluSpots modified histone tail peptide array.

**Additional file 3.** Interaction of the D3PWWP-M8Chromo double-HiMID with double modified H3-GST peptides.

## Abbreviations

ChIP: chromatin immunoprecipitation
CIDOP: chromatin interacting domain precipitation
GO: gene ontology
HiMID: histone modification interacting domain
PKMT: protein lysine methyltransferase
PTM: post-translational modification
qPCR: quantitative PCR
ZNF: zinc finger
