# Supplementary material for: Application of dual reading domains as novel reagents in chromatin biology reveals a new H3K9me3 and H3K36me2/3 bivalent chromatin state
Source: Epigenetics Chromatin. 2017 Sep 25;10:45. doi: 10.1186/s13072-017-0153-1 (PMC5613355; doi:10.1186/s13072-017-0153-1)
Supplement: Supplementary file 1 — Additional file 1: Figure S1. Analysis of the coexistence of H3K9me3 and H3K36me2/3 in one H3 molecule by mass spectrometry. Figure S2. Control experiments related to Fig. 2. Figure S3. Quality control of double peptide spot arrays. Figure S4. Definition of chromatin states in HepG2 cells based on H3K9me3 and H3K36me2/3 CIDOP-seq signals using 1 kb bins. Figure S5. Definition of chromatin states based on Ernst et al. 2011. Figure S6. Annotated sequence of the D3PWWP-M8Chromo double domain. Table S1. GO analysis of low expression gene clusters shown in Fig. 6c. Table S2. GO analysis of the clusters of genes regulated by weak enhancers-2 shown in Fig. 7b. Table S3. GO analysis of the clusters of genes regulated by strong enhancers-2 shown in Fig. 7c. Table S4. Sequences of primers used in Fig. 5 for CIDOP-qPCR. Table S5. Sequences of primers used in Fig. 8b for CIDOP-qPCR. [file 13072_2017_153_MOESM1_ESM.pdf]

Application of dual reading domains as novel reagents in chromatin biology reveals a new H3K9me3 and H3K36me2/3 bivalent chromatin state

Rebekka Mauser, Goran Kungulovski, Corinna Keup, Richard Reinhardt and Albert Jeltsch

Supplemental Figures

Figure S1. Analysis of the co-existence of H3K9me3 and H3K36me2/3 in one H3 molecule by mass spectrometry.

Figure S2. Control experiments related to Figure 2.

Figure S3. Quality control of double peptide spot arrays.

Figure S4. Additional controls related to Fig. 4D.

Figure S5. Definition of chromatin states based on Ernst et al., 2011.

Figure S6. Annotated sequence of the D3PWWP-M8Chromo double domain.

Supplemental Tables

Table S1. GO analysis of low expression gene clusters shown in Figure 6C.

Table S2. GO analysis of the clusters of genes regulated by weak enhancers-2 shown in Figure 7B.

Table S3. GO analysis of the clusters of genes regulated by strong enhancers-2 shown in Figure 7C.

Table S4. Sequences of primers used in Figure 5 for CIDOP-qPCR.

Table S5. Sequences of primers used in Figure 8B for CIDOP-qPCR.

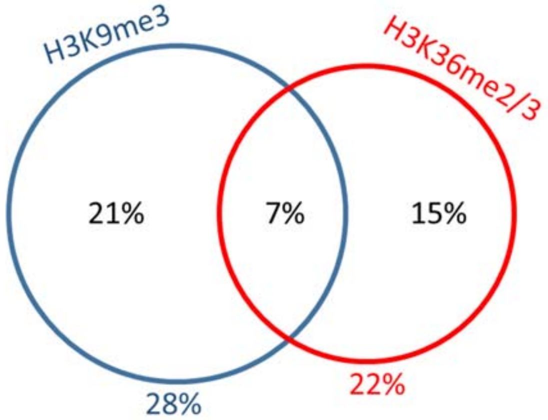

Figure S1. Analysis of the co-existence of H3K9me3 and H3K36me2/3 in one H3 molecule by mass spectrometry (Young et al., 2009). Percent values refer to the overall intensities observed on H3 tails carrying H3K9me3 (blue label) and H3K36me2/3 (red label). The black labels refer to H3K9me3-only, H3K9me3-K36me2/3 and H3K36me2/3-only states.

Reference

Young, N.L., DiMaggio, P.A., Plazas-Mayorca, M.D., Baliban, R.C., Floudas, C.A. and Garcia, B.A. (2009) High throughput characterization of combinatorial histone codes. Molecular & cellular proteomics: MCP, 8, 2266-2284.

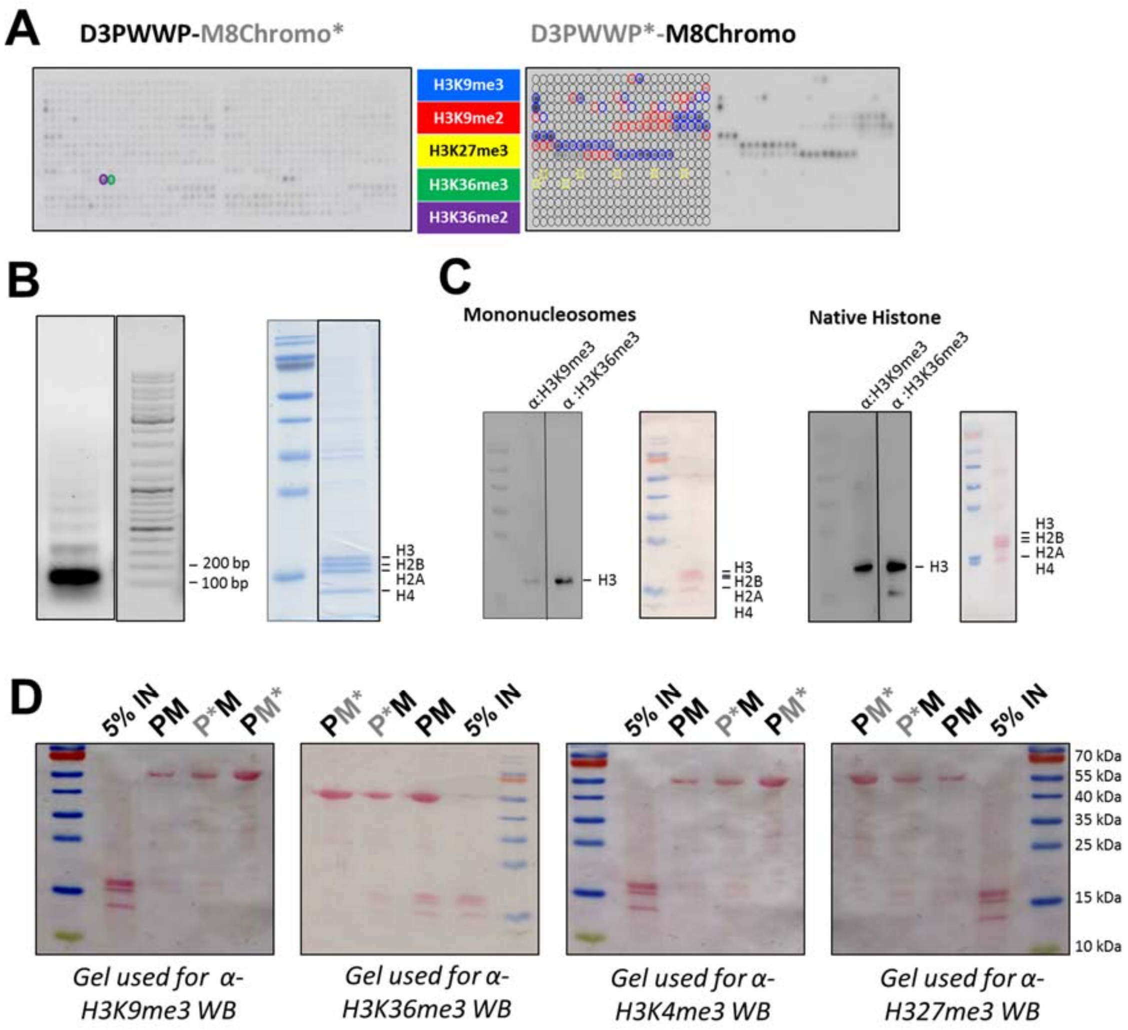

Figure S2. Control experiments related to Figure 2. **A)** Peptide array specificity profiling of D3PWWP-M8Chromo variants. The peptide spots are annotated between the two arrays and the color code denotes the presence of the designated histone PTM in different combinations. The D3PWWP*-M8Chromo displayed binding only to H3K9me3 and H3K27me3 peptides, while the D3PWWP-M8Chromo* displayed binding to H3K36me2 and H3K36me3 peptides. **B)** Quality analysis of mononucleosomes. Gel red stained agarose gel of mononucleosomal DNA (left) and Coomassie BB stained SDS-PAGE of the protein content of the mononucleosomes (right) used in this study (in both cases the marker and the sample belong to the same gel). **C)** Quality analysis of mononucleosomes and native histones. In the left panels, western blot analyses with H3K9me3 and H3K36me3 antibodies are shown documenting the presence of both modifications. In the right panels, the corresponding Ponceau S stained images are shown (in all cases the marker and the sample belong to the same gel). **D)** Ponceau S stained images of the WB membranes shown in Figure 2D. The bands at 54 kDa correspond to the GST tagged double-HiMID proteins.

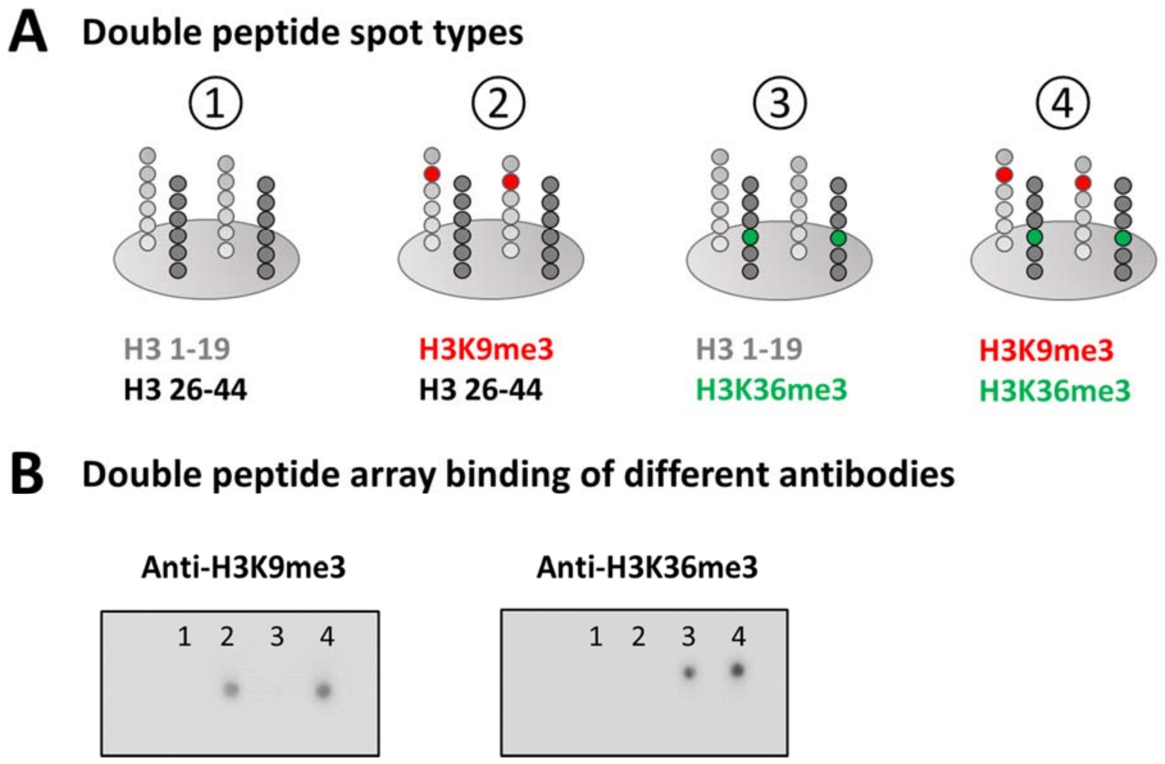

Figure S3. Quality control of double peptide spot arrays. **A)** Design of the double peptide arrays. Unmodified, K9me3 and K36me3 modified H3 (1-19) and H3 (26-44) peptides were mixed in all four possible combinations and spotted on a glass slide. **B)** Binding of H3K9me3 and H3K36me3 antibodies to the double peptide arrays, confirming the presence of H3K9me3 in spots 2 and 4 and H3K36me3 in spots 3 and 4. This information is related to Figure 3.

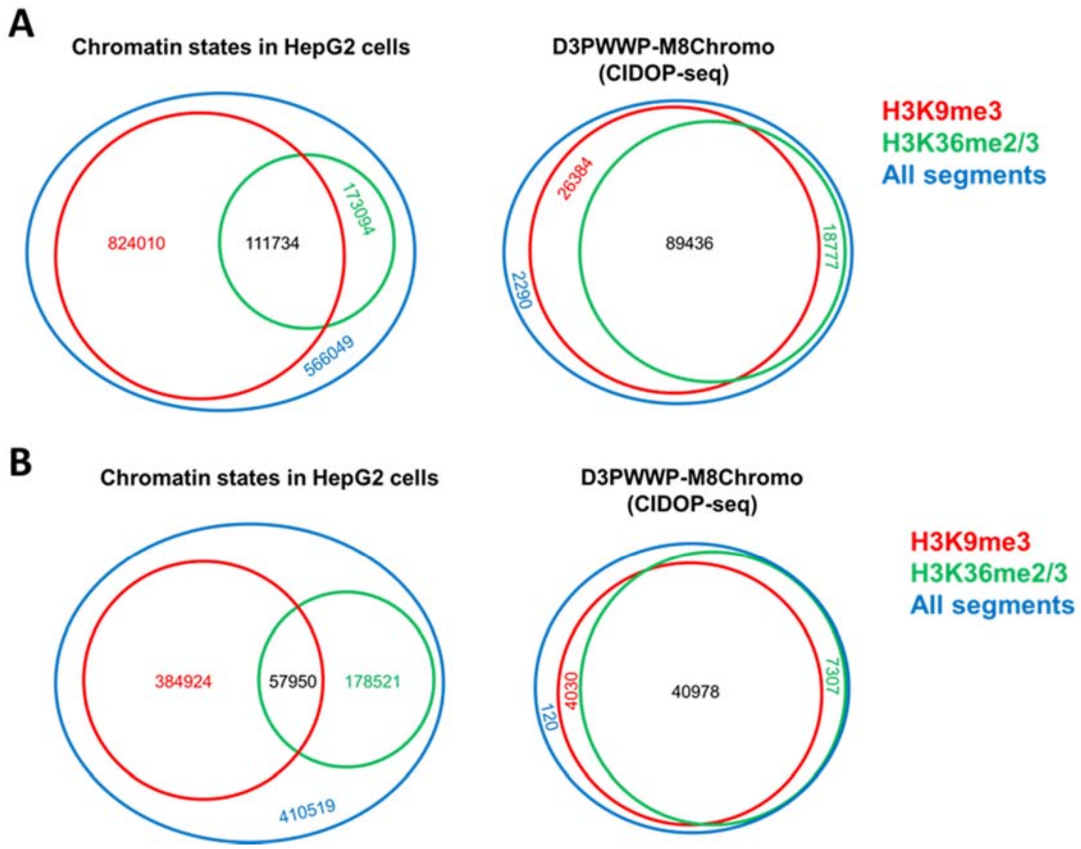

Figure S4. Additional controls related to Fig. 4D. **A)** Analysis of chromatin states in HepG2 cells based on H3K9me3 and H3K36me2/3 CIDOP-seq signals using 1 kb bins for chromosome 1-9. Similarly as described in the text for Figure 4D, we defined four chromatin states based on the distribution of H3K9me3 (merged MPP8 Chromo and D3PWWP*-M8Chromo data) and H3K36me2/3 (Dnmt3a PWWP data): without H3K9me3 and H3K36me2/3, H3K36me2/3-only, H3K9me3-only and overlap of H3K36me2/3 and H3K9me3. The red circle indicates the number of regions with H3K9me3 signal, the green circle indicates regions with H3K36me2/3 signal and the blue circle indicates all regions. Red numbers refer to only H3K9me3 regions, green number to only H3K36me3 region, black numbers to regions with both signals, blue number to regions without both signals. The distribution of chromatin states bound by D3PWWP-M8Chromo shows a strong and selective enrichment of H3K9me3-H3K36me2/3 dual state with 80% recovery of all H3K9me3-H3K36me2/3 overlap regions. In contrast, the H3K9me3-only and H3K36me2/3-only regions were only weakly recovered. **B)** Analysis of chromatin states in HepG2 cells and in D3PWWP-M8Chromo pulldown based on H3K9me3 and H3K36me2/3 CIDOP-seq signals using 3 kb bins using more stringent signal thresholds than in Fig. 4D. The thresholds used in Fig. 4D correspond to a false discovery rate (FDR) of the detection of overlap between D3PWWP-M8Chromo pulldown and regions containing H3K9me3 and H3K36me2/3 modifications of 15%, the more stringent thresholds applied here correspond to a FDR of 5%. As in Fig. 4D and panel A of this figure, the distribution of chromatin states bound by D3PWWP-M8Chromo shows a strong and selective enrichment of H3K9me3-H3K36me2/3 dual state with 78% recovery of all H3K9me3-H3K36me2/3 overlap regions. In contrast, the H3K9me3-only and H3K36me2/3-only regions were only weakly recovered.

	H3K36me3	H3K4me1	H3K4me2	H3K4me3	H3K27ac	H3K9ac	DNA accecibility	Transcription levels
SE-1	15	96	99	75	97	86	23.1	1.3
SE-2	10	88	57	5	84	25	13.6	1.4
WE-1	1	58	75	8	6	5	11.9	1.1
WE-2	2	56	3	0	6	2	5.1	1.3
Weak transcribed	3	0	0	0	0	0	0.3	1.9

Figure S5. Definition of chromatin states based on Ernst et al. (2011). Strong enhancers-1 (SE-1, designated as strong enhancer-4 by Ernst et al.) are defined by low levels of H3K36me3, high levels of H3K4me1, H3K4me2, H3K4me3, H3K27ac, H3K9ac and high DNA accessibility. Strong enhancers-2 (SE-2, designated as strong enhancers-5 by Ernst et al.) are defined by high levels of H3K4me1 and H3K27ac, medium levels of H3K4me2 and no H3K4me3, low levels of H3K36me3 and H3K9ac and a two-fold lower DNA accessibility in comparison to SE-1. Weak enhancers-1 (WE-1, designated as weak/poised enhancer-6 in by Ernst et al.) are defined by medium levels of H3K4me1, high levels of H3K4me2 and almost no H3K36me3, H3K4me3, H3K9ac and H3K27ac, with DNA accessibility similar to strong enhancers-2. Weak enhancers-2 (WE-2, designated as weak/poised enhancer-7 in by Ernst, et al.) are defined by medium levels of H3K4me1 and almost no H3K36me3, H3K4me2, H3K4me3, H3K9ac and H3K27ac, with DNA accessibility two fold lower than SE-2 or WE-1. Weak transcribed states are defined by little H3K36me3, no H3K4me1/2/3, no H3K27ac, no H3K9ac, low DNA accessibility and weak transcription. For histone PTMs each number and the intensity of red shading denotes the frequency with which a given mark is found at genomic positions corresponding to the chromatin state. Numbers for DNA accessibility and transcript level represent fold enrichment [1]. This information is related to Figure 7A.

Reference

1. Ernst J, Kheradpour P, Mikkelsen TS, Shoresh N, Ward LD, Epstein CB, Zhang X, Wang L, Issner R, Coyne M, et al: **Mapping and analysis of chromatin state dynamics in nine human cell types.** *Nature* 2011, **473:**43-49.

MSPILGYWKIKGLVQPTRLLLEYLEEKYEEHLYERDEGDKWRNKKFELGLEFPNLPYYIDGDVKLTQSMAIIRYIADK
HNMLGGCPKERAEISMLEGAVLDIRYGVSRIAYSKDFETLKVDFLSKLPEMLKMFEDRLCHKTYLNGDHVTHPDFM
LYDALDVVLYMDPMCLDAFPKLVCFKKRIEAIPQIDKYLKSSKYIAWPLQGWQATFGGGDHPPKSDLEVLFQGPLG
SPGILGSEYEDGRGFGIGELVWGKLRGFSWWPGRIVSWWMTGRSRAAEGTRWVMWFGDGKFSVVCVEKLMP
LSSFCSAFHQATYNKQPMYRKAIYEVLQVASSRAGKLFPACHDSDESDSGKAVEVQNKQMIEWALGGFQPSGPK
GLEPPLERPHRDVFEVEKILDMKTEGGKVLYKVRWKGYTSDDDTWEPEIHLEDCKEVLLEFRKKIAEPGSTRAAAS*

GST –TAG

PreScision protease site

Dnmt3a PWWP domain

Putative natural linker taken from Dnmt3a

MPP8 Chromo domain

Figure S6. Annotated sequence of the D3PWWP-M8Chromo double domain.

Table S1. GO analysis of low expression gene clusters shown in Figure 6C.

Cluster	GO category – biological process	p-value
1	long-chain fatty-acyl-CoA metabolic process	1.07E-06
	regulation of anti-apoptosis	2.27E-06
	fatty-acyl-CoA metabolic process	5.81E-06
	glycerolipid biosynthetic process	8.36E-06
	positive regulation of heart rate	8.37E-06
	anti-apoptosis	4.93E-05
	positive regulation of anti-apoptosis	9.09E-05
	acyl-CoA metabolic process	1.34E-04
	labyrinthine layer morphogenesis	1.91E-04
	embryonic placenta morphogenesis	1.99E-04
2	triglyceride biosynthetic process	3.39E-06
	neutral lipid biosynthetic process	3.90E-06
	venous blood vessel development	9.10E-06
	retina vasculature development in camera-type eye	1.40E-05
	lymphangiogenesis	1.64E-05
	positive regulation of triglyceride biosynthetic process	1.79E-05
	glycerolipid biosynthetic process	3.46E-05
	response to endoplasmic reticulum stress	6.16E-05
	regulation of triglyceride biosynthetic process	6.28E-05
	lymph vessel development	1.18E-04
	rRNA transcription	1.69E-04
3	base-excision repair	2.54E-06
	2'-deoxyribonucleotide metabolic process	6.27E-06
	centrosome organization	6.35E-06
	cilium assembly	1.20E-05
	microtubule organizing center organization	1.59E-05
	centrosome cycle	1.83E-05
	deoxyribonucleotide metabolic process	2.93E-05
	regulation of intracellular pH	4.20E-05
	regulation of cellular pH	5.88E-05
	pyrimidine deoxyribonucleotide metabolic process	7.49E-05
	G2/M transition of mitotic cell cycle	8.92E-05
4	SCF-dependent proteasomal ubiquitin-dependent protein catabolic process	8.24E-06
	peripheral nervous system neuron differentiation	7.36E-05
	mitochondrion localization	7.46E-05
	tRNA modification	2.25E-04
	spinal cord association neuron differentiation	4.22E-04
	hippo signaling cascade	4.76E-04
	positive regulation of CREB transcription factor activity	5.56E-04

	peptidyl-histidine modification	0.00122
	dorsal spinal cord development	0.00166
	melanosome organization	0.00178
**5**	lung development	2.15E-05
	regulation of nitric-oxide synthase activity	2.67E-05
	respiratory tube development	2.71E-05
	regulation of monooxygenase activity	1.09E-04
	embryonic digit morphogenesis	1.20E-04
	respiratory system development	1.20E-04
	ncRNA processing	1.64E-04
	regulation of cholesterol metabolic process	1.75E-04
	mesodermal cell fate commitment	2.20E-04
	multicellular organism growth	5.07E-04
	mesodermal cell differentiation	6.10E-04
	epithelial tube branching involved in lung morphogenesis	6.22E-04
	embryonic limb morphogenesis	6.66E-04
	tube development	7.13E-04
**6**	hypothalamus development	5.93E-07
	DNA-dependent DNA replication initiation	8.40E-07
	bile acid metabolic process	1.39E-06
	M/G1 transition of mitotic cell cycle	2.57E-06
	bile acid biosynthetic process	4.40E-06
	S phase of mitotic cell cycle	8.88E-06
	cellular response to epidermal growth factor stimulus	8.88E-06
	indolalkylamine metabolic process	1.11E-05
	progesterone metabolic process	1.40E-05
	androgen metabolic process	2.24E-05
	S phase	2.29E-05
**7**	zinc ion binding	9.06E-12
	positive regulation of chondrocyte differentiation	3.90E-04
	regulation of cartilage development	5.08E-04
	osteoblast development	6.54E-04
	protein autoubiquitination	0.00144
	embryonic skeletal system morphogenesis	0.00154
	negative regulation of catabolic process	0.00189
	negative regulation of protein catabolic process	0.00197
	negative regulation of osteoblast differentiation	0.0022
	protoporphyrinogen IX metabolic process	0.00271
**8**	negative regulation of smoothened signaling pathway	6.62E-07
	membrane depolarization	3.15E-04
	visual learning	3.64E-04
	regulation of smoothened signaling pathway	4.54E-04

	midbrain-hindbrain boundary development	4.95E-04
	negative regulation of sequence-specific DNA binding transcription factor activity	5.89E-04
	rostrocaudal neural tube patterning	6.30E-04
	cerebellar cortex formation	6.46E-04
	visual behavior	6.98E-04
	positive regulation of triglyceride biosynthetic process	7.06E-04
	triglyceride biosynthetic process	8.42E-04
	neutral lipid biosynthetic process	8.69E-04
**9**	chromatin remodeling at centromere	3.23E-05
	histone exchange	4.91E-05
	lung epithelial cell differentiation	5.14E-05
	centromere complex assembly	7.18E-05
	lung cell differentiation	7.61E-05
	ATP-dependent chromatin remodeling	1.10E-04
	acrosome reaction	2.91E-04
	organelle localization	4.18E-04
	DNA replication-independent nucleosome assembly	9.53E-04
	protein-DNA complex assembly	0.00118
	mitotic prometaphase	0.00119
	chromosome segregation	0.00128
**10**	cellular response to amine stimulus	1.04E-05
	cellular response to organic nitrogen	1.70E-05
	dichotomous subdivision of an epithelial terminal unit	1.82E-04
	pyrimidine nucleobase metabolic process	2.36E-04
	cellular response to amino acid stimulus	3.96E-04
	cellular response to cAMP	5.27E-04
	cellular biogenic amine biosynthetic process	6.47E-04
	response to iron ion	8.34E-04
	modification by symbiont of host morphology or physiology	8.55E-04
	pigmentation	8.80E-04
	multicellular organismal movement	0.00112
	amine biosynthetic process	0.00117
	cellular response to acid	0.00128

Table S2. GO analysis of the clusters of genes regulated by weak enhancers-2 shown in Figure 7B.

**Cluster**	**GO category – biological process**	**p-value**
**4**	ribonucleoside bisphosphate metabolic process	4.34E-06
	xenobiotic metabolic process	8.14E-06
	response to xenobiotic stimulus	9.23E-06
	glycoside metabolic process	3.65E-05
	bile acid biosynthetic process	6.88E-05
	primary alcohol metabolic process	1.55E-04
	ectodermal placode formation	1.62E-04
	cellular hormone metabolic process	2.13E-04
	nucleoside bisphosphate metabolic process	2.41E-04
	cellular polysaccharide biosynthetic process	3.08E-04
**5**	microtubule anchoring	5.24E-05
	regulation of microtubule-based process	1.04E-04
	induction of apoptosis by extracellular signals	1.72E-04
	extrinsic apoptotic signaling pathway	2.34E-04
	tolerance induction	3.11E-04
	DNA modification	5.53E-04
	erythrocyte maturation	8.28E-04
	enzyme binding	8.38E-04
	negative regulation of protein complex disassembly	8.43E-04
	protein depolymerization	0.00121
**6**	negative regulation of bone mineralization	3.01E-05
	negative regulation of biomineral tissue development	3.06E-05
	positive regulation of endothelial cell proliferation	4.61E-05
	nuclear export	7.34E-05
	cellular carbohydrate metabolic process	1.33E-04
	protein export from nucleus	1.98E-04
	oligodendrocyte differentiation	6.92E-04
	interleukin-1-mediated signaling pathway	7.24E-04
	protein K63-linked deubiquitination	7.66E-04
	integrin-mediated signaling pathway	8.81E-04
	hemoglobin metabolic process	8.83E-04
	regulation of protein kinase A signaling cascade	9.80E-04
**7**	xenobiotic metabolic process	1.91E-07
	response to xenobiotic stimulus	2.21E-07
	ethanol oxidation	3.42E-06
	oxidation-reduction process	1.68E-05
	initiation of viral infection	1.78E-05
	terpenoid metabolic process	3.13E-05
	vitamin metabolic process	3.91E-05
	vitamin A metabolic process	8.82E-05

	ethanol metabolic process	9.39E-05
	glycerolipid catabolic process	1.60E-04
**8**	response to lipid	1.07E-06
	positive regulation of collagen metabolic process	4.49E-06
	regulation of cell cycle process	6.21E-06
	protein-DNA complex assembly	9.39E-06
	regulation of collagen biosynthetic process	1.97E-05
	regulation of collagen metabolic process	2.02E-05
	positive regulation of multicellular organismal metabolic process	2.97E-05
	protein-DNA complex subunit organization	3.88E-05
	response to fatty acid	4.02E-05
	negative regulation of T cell differentiation	4.90E-05
	pantothenate metabolic process	4.93E-05

Table S3. GO analysis of the clusters of genes regulated by strong enhancers-2 shown in Figure 7C.

**Cluster**	**GO category – biological process**	**p-value**
**5**	regulation of protein localization	2.06E-05
	negative regulation of protein transport	3.22E-05
	glycogen metabolic process	1.24E-04
	cellular glucan metabolic process	1.24E-04
	bile acid biosynthetic process	1.25E-04
	vesicle transport along microtubule	1.82E-04
	monocarboxylic acid metabolic process	2.96E-04
	regulation of reactive oxygen species metabolic process	3.27E-04
	negative regulation of intracellular protein transport	3.94E-04
	DNA damage response, signal transduction by p53 class mediator resulting in cell cycle arrest	4.44E-04
	signal transduction involved in DNA integrity checkpoint	4.44E-04
**6**	response to lipid	5.51E-08
	cartilage development involved in endochondral bone morphogenesis	4.04E-07
	DNA strand elongation involved in DNA replication	1.78E-06
	connective tissue development	1.28E-05
	peptide cross-linking	2.15E-05
	response to corticosteroid stimulus	3.50E-05
	digestion	3.70E-05
	triglyceride catabolic process	3.90E-05
	neutral lipid catabolic process	3.90E-05
	skeletal system morphogenesis	4.26E-05
**7**	primary alcohol metabolic process	1.04E-06
	ribonucleoside bisphosphate metabolic process	1.40E-06
	xenobiotic metabolic process	3.40E-06
	response to xenobiotic stimulus	3.77E-06
	sulfation	6.00E-06
	ethanol oxidation	1.11E-05
	cellular hormone metabolic process	2.08E-05
	activation of MAPKK activity	2.69E-05
	vitamin A metabolic process	3.17E-05
	cellular aldehyde metabolic process	5.94E-05
	nucleoside bisphosphate metabolic process	6.95E-05

Table S4. Sequences of primers used in Figure 5 for CIDOP-qPCR and sequential CIDOP/ChIP.

Target	Name	Position	Sequence
H3K9me3 and H3K36me3	9	Chr 4, Position: 455037-455147 (111 bp)	FP CTAGAGCCCAGGCTGTTTTG<br>RP AAGCAGATTGCAGTGGGAAG
	10	Chr 19, Position: 36449278-36449371 (94 bp)	FP GCATTGCCACATTCCTTACA<br>RP GCGGGAAGGCCTTTAGTAGT
	11	Chr 19, Position: 37739671-37739765 (95 bp)	FP TGGTTTTTCGCCAGTATGAA<br>RP GTGGGAAGCCGTATGAATGT
	12	Chr 19, Position: 40015195-40015290 (96bp)	FP GTGGGAAGGCCTTTATTCGT<br>RP ACGTCTTCCCACATTCCGTA
H3K36me3	5	Chr 12, Position: 6645729-6645847 (119 bp)	FP CAATGACCCCTTCATTGACC<br>RP GGGGGAATACGTGAGGGTAT
	6	Chr 9, Position: 100402514-100402612 (99 bp)	FP TGCTCCTTTTTCCCATCTTTT<br>RP GCAAAACCAAGTCGAATGCT
	7	Chr 19, Position: 38660862-38660965 (104bp)	FP AGTGATTGGGAATGGCTTTG<br>RP AACAGCTGAAATGCCACCTC
	8	Chr 19, Position: 41272007-41272112 (106 bp)	FP GGGAAAAGGGACCAGAAAAG<br>RP GGTGCTCCTTGCTTCATCTC
H3K9me3	1	Chr 5, Position: 22891711-22891829 (119 bp)	FP TGCATGATGTTTTCCTCAGC<br>RP ATCTTGCGCAAATGCTCTG
	2	Chr 19, Position: 37625187-37625309 (123 bp)	FP TTGTCACCACTGTCCAGGAA<br>RP CAGCTGCCTCAGAGACACAC
	3	Chr 5, Position: 22528920-22529032 (113 bp)	FP AGAACACCATGGACCACCAG<br>RP TTTCTGAATTGGTTCTGGGTTT
	4	Chr 19, Position: 40954465-40954565 (101 bp)	FP TTCACAGAAACTGCCACTGC<br>RP GCAGATGAGAAGGCAAGGAA

Table S5. Sequences of primers used in Figure 8B for CIDOP-qPCR.

Target	Name	Position	Sequence
ZFN274 binding sites with H3K9me3-H3K36me3 signal	20	Chr 19, Position: 52554314-52554418 (105 bp)	FP:TAATTCACAGCTGGCACGAC RP: TGGCATAGAAGGGATGACCT
	21	Chr 19, Position: 52840919-52841019 (101 bp)	FP: TCTGATGTTGTGCCAGGTGT RP: CCTGGAAAGACACAGGAGGA
	22	Chr 19, Position: 52949774-52949856 (83 bp)	FP:TGATTTGCAATGGTTGTAGCA RP AAGCAATCCATGGGTGTAGG
	24	Chr 19, Position: 53410202-53410301 (100 bp)	FP: TCAAAAAGCAAAGCTTGCAC RP: GAGGCTTGATTTGCGACTGT
	25	Chr 19, Position: 53456346-53456442 (97 bp)	FP GACCTTCAGTCGGACGTCAT RP CACACGGAAAGCTTTGTCAC
Control region	23	Chr 19, Position: 53305130-53305236 (107 bp)	FP: TTCCTTCCTGCATGAGATCC RP: GAGTGCTTCCCCCAGATTTT
	26	Chr 19, Position: 53879224-53879313 (90 bp)	FP:CTATCCTTCGCCCTAGGTCAC RP: GGGAATCCGAGTCCTCTTGT
