# Supplementary material for: Application of dual reading domains as novel reagents in chromatin biology reveals a new H3K9me3 and H3K36me2/3 bivalent chromatin state
Source: Epigenetics Chromatin. 2017 Sep 25;10:45. doi: 10.1186/s13072-017-0153-1 (PMC5613355; doi:10.1186/s13072-017-0153-1)
Supplement: Supplementary file 3 — Additional file 3. Interaction of the D3PWWP-M8Chromo double-HiMID with double modified H3-GST peptides. [file 13072_2017_153_MOESM3_ESM.pdf]

Application of dual reading domains as novel reagents in chromatin biology reveals a new H3K9me3 and H3K36me2/3 bivalent chromatin state

Rebekka Mauser, Goran Kungulovski, Corinna Keup, Richard Reinhardt and Albert Jeltsch

Interaction of the D3PWWP-M8Chromo double HiMID with double modified H3-GST peptides

The interaction of D3PWWP-M8Chromo could be either based on the recognition of two separate histone tails harboring H3K36me2/3 or H3K9me3 or due to the presence of the H3K36me2/3 and H3K9me3 marks on the same H3 tail or a combination of both. To discriminate these binding modes, we generated a recombinant H3 fragment consisting of the first 60 amino acids N-terminally fused to GST (H3-GST). Using the methyl-lysine analogue technology [1], we generated H3-GST proteins with a trimethyllysine analogue at position 9 (H3K9$_C$me3), position 36 (H3K36$_C$me3) or at both sites (H3K9$_C$me3-H3K36$_C$me3), with similarly high (almost 100%) efficiency of conversion as shown by mass spectrometry (Additional file 2, Figure 1A). Then, these GST tagged H3 proteins were used as bait for pulldown reactions with maltose binding protein (MBP) tagged D3PWWP-M8Chromo and its binding pocket mutants (Additional file 2, Figure 1b), using stringent washing conditions for D3PWWP-M8Chromo and D3PWWP*-M8Chromo, but relaxed washing conditions for D3PWWP-M8Chromo*. As expected D3PWWP-M8Chromo* showed binding to H3K36$_C$me3 and H3K9$_C$me3-H3K36$_C$me3 and D3PWWP*-M8Chromo bound H3K9$_C$me3 and H3K9$_C$me3-H3K36$_C$me3, indicating that both individual domains interact with the methyllysine analog modified H3 tail.

Like D3PWWP*-M8Chromo, the double-HiMID exhibited strong binding to H3K9$_C$me3. As expected binding to H3K36$_C$me3 was much weaker under the same stringent washing conditions, indicating that the binding of the M8Chromo part to H3K9$_C$me3 is the primary interaction. If binding of both domains to one double modified peptide were possible, the double domain should bind more strongly to the double modified peptide than to single modified H3K9me3 modified peptides, because of the additional binding of the PWWP domain to K36$_c$me3. However, such an improved binding of the double-HiMID to H3K9$_C$me3-H3K36$_C$me3 was not observed. The lack of improved binding of D3PWWP-M8Chromo to double modified H3K9$_C$me3-H3K36$_C$me3 H3-GST when compared to single modified H3K9$_C$me3 H3-GST, suggests that the domain is not able to bind both marks *in cis*. Probably, this interaction is sterically precluded, because the domains are not in a position that allows for continuous binding to one H3 peptide by the M8Chromo and the D3PWWP domain next to each other. Hence, the binding of the D3PWWP-M8Chromo fusion domain to H3K9me3-H3K36me2/3 double modified mononucleosomes observed in Figure 2C and in the following sections of this manuscript likely occurs *in trans.* This conclusion is also in line with the results of the double peptide array, were synergistic binding to both modifications presented on separate peptides was observed. The weaker binding of D3PWWP-M8Chromo to double modified H3K9$_C$me3-H3K36$_C$me3 H3-GST as compared to only H3K9$_C$me3 H3-GST modified might be explained by the fact that in this case an averaged binding affinity to H3K9$_C$me3 or H3K36$_C$me3 was detected.

Methods

The first 60 amino acids of human histone H3 were cloned with C-terminal GST tag with Gibson assembly into a pGEX-6p-2 vector variant in which all cysteines in GST were replaced with serine. Afterwards, the targeted lysine(s) in histone H3 were replaced with cysteine and alkylated with (2-bromoethyl) trimethylammonium bromide to the respective trimethyl analog as described [1]. The efficiency of the conversion was verified by MALDI-TOF mass spectrometry. For pull-downs, 25 µg of MLA modified H3-GST were incubated with 0.5 µM of MBP-tagged HiMID overnight in DP buffer [16.7 mM Tris-HCl, 167 mM NaCl, 1.1% Triton X-100, 1.2 mM EDTA supplemented with protease inhibitors (cOmplete ULTRA Tablets, Mini, EDTA-free, Easy pack from Roche)] at +4°C with rotation. Next, the bound complexes were immobilized with 20-40 µl glutathione sepharose 4B beads (GE Healthcare) for 2 hours at +4°C with rotation, washed three times with CIDOP buffers [1x 1 ml low salt buffer (20 mM Tris-HCl pH 8, 150 mM NaCl, 1% Triton X-100, 2 mM EDTA, 0.1 % SDS), 1x 1 ml high salt buffer (20 mM Tris-HCl pH 8, 500 mM NaCl, 1% Triton X-100, 2 mM EDTA, 0.1 % SDS), 1 x 1 ml LiCl buffer (10 mM Tris-HCl pH 8, 250 mM LiCl, 1% Nonident-P40, 1 mM EDTA, 1% Sodium deoxycholate)] (for the D3PWWP-M8Chromo* three times with PB200 buffer (50mM Tris-HCl pH 8, 200mM NaCl, 1mM EDTA, 2mM DTT, 0,5% NP-40) and twice with TE buffer (10 mM Tris-HCl pH 8, 1 mM EDTA), each for 5 min with rotation followed by centrifugation for 2 min at +4°C, 2000 rcf. The precipitated histones were eluted with LAP, electrophoresed on an 18% SDS-PA gel, transferred on a nitrocellulose membrane and probed with anti-MBP antibody (New England Biolabs, E8032).

Reference

1. Simon MD, Chu F, Racki LR, de la Cruz CC, Burlingame AL, Panning B, Narlikar GJ, Shokat KM: **The site-specific installation of methyl-lysine analogs into recombinant histones.** *Cell* 2007, **128:**1003-1012.

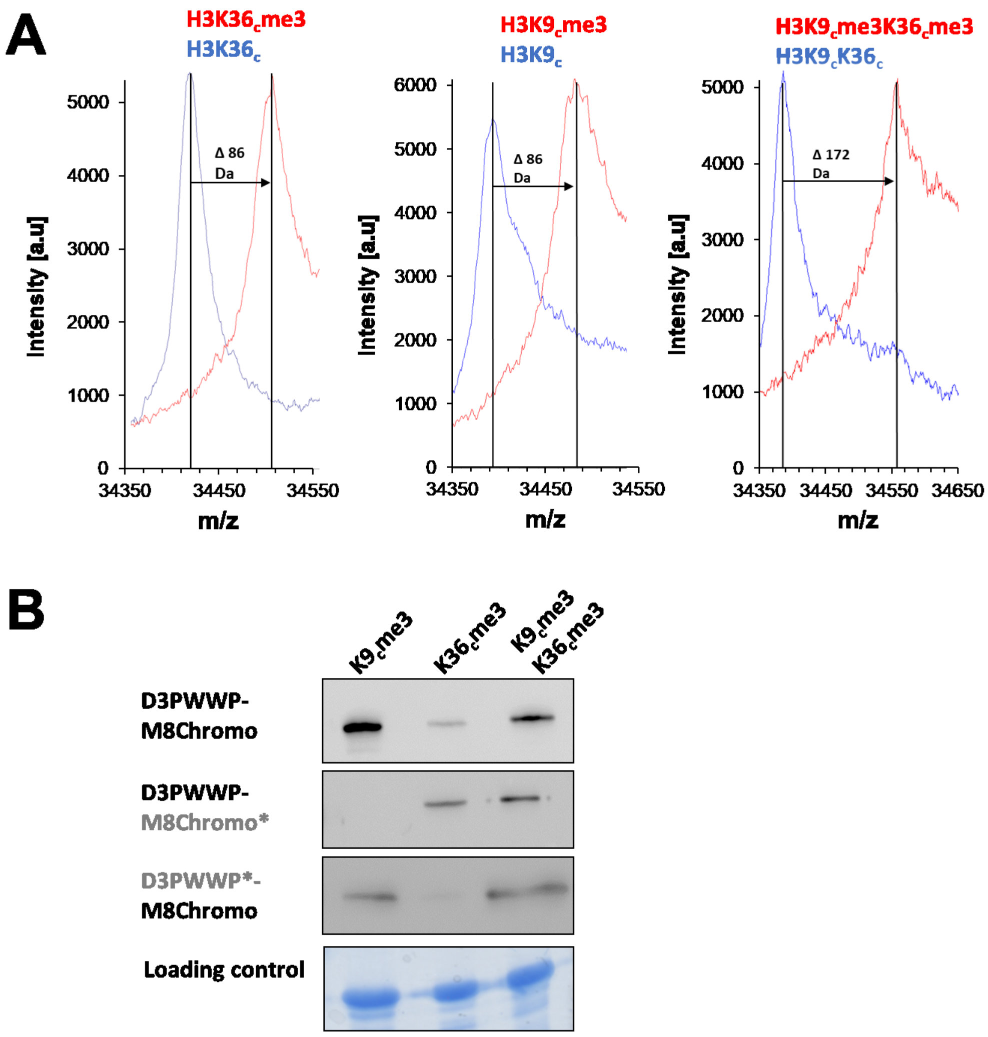

Additional file 3 figure. Interaction of the D3PWWP-M8Chromo double HiMID with double modified H3-GST peptides. **A)** Quality control of the H3-GST proteins containing methyllysine analogs. Lysine 9, 36 or both from H3-GST were replaced by cysteine (H3K9C, H3K36C, and H3K9C/K36C) and subsequently converted to the respective trimethyl analog ($H3K9_Cme3$, $H3K36_Cme3$, and $H3K9_Cme3K36_Cme3$). Mass spectrometry analyses indicate a high efficiency of conversion (blue before conversion, red after conversion) of methyllysine analogs, because the unconverted substrate was no longer detectable after conversion. The theoretical masses are: H3K9C 34417 Da, H3K36C 34417 Da, H3K9C/K36C 34392 Da, $H3K9_Cme3$ 34503 Da, $H3K36_Cme3$ 34503 Da and $H3K9_Cme3K36_Cme3$ 34564 Da. In all cases the detected masses were corresponding to the expected mass within the range of error of this experiment. The expected mass shift by one alkylation reaction is +86 Da. This information is related to Figure 4. Schematic representation of the principles of recognition of the co-existence of two histone PTMs on mononucleosomes by fused double reading domains interacting with the two marks on one H3 tail (in “cis”) or on separate H3 tails (in “trans”). **B)** Pull-down of MBP-D3PWWP-M8Chromo and its variants with H3-GST trimethyl analogs at positions 9, 36 or both, detection with anti-MBP antibody. For D3PWWP-M8Chromo and D3PWWP*-M8Chromo stringent (CIDOP) washing conditions were used, for D3PWWP-M8Chromo* PB200 buffer was used for washing. The Coomassie BB stained image represents a loading control of modified H3-GST.
